# Transcellular chaperone signaling is an intercellular stress-response distinct from the HSF-1–mediated heat shock response

**DOI:** 10.1371/journal.pbio.3001605

**Published:** 2023-02-13

**Authors:** Jay Miles, Sarah Townend, Dovilė Milonaitytė, William Smith, Francesca Hodge, David R. Westhead, Patricija van Oosten-Hawle

**Affiliations:** School of Molecular and Cell Biology & Astbury Centre for Structural Molecular Biology, Faculty of Biological Sciences, University of Leeds, Leeds, United Kingdom; U of Michigan, UNITED STATES

## Abstract

Organismal proteostasis is maintained by intercellular signaling processes including cell nonautonomous stress responses such as transcellular chaperone signaling (TCS). When TCS is activated upon tissue-specific knockdown of *hsp-90* in the *Caenorhabditis elegans* intestine, heat-inducible *hsp-70* is induced in muscle cells at the permissive temperature resulting in increased heat stress resistance and lifespan extension. However, our understanding of the molecular mechanism and signaling factors mediating transcellular activation of *hsp-70* expression from one tissue to another is still in its infancy. Here, we conducted a combinatorial approach using transcriptome RNA-Seq profiling and a forward genetic mutagenesis screen to elucidate how stress signaling from the intestine to the muscle is regulated. We find that the TCS-mediated “gut-to-muscle” induction of *hsp-70* expression is suppressed by HSF-1 and instead relies on *transcellular-X-cross-tissue (txt)* genes. We identify a key role for the PDZ-domain guanylate cyclase *txt-1* and the homeobox transcription factor *ceh-58* as signaling hubs in the stress receiving muscle cells to initiate *hsp-70* expression and facilitate TCS-mediated heat stress resistance and lifespan extension. Our results provide a new view on cell-nonautonomous regulation of “inter-tissue” stress responses in an organism that highlight a key role for the gut. Our data suggest that the HSF-1–mediated heat shock response is switched off upon TCS activation, in favor of an intercellular stress-signaling route to safeguard survival.

## Abstract 2

The transcellular activation of Hsp70 chaperone from one tissue to another is poorly understood. A study in the nematode Caenorhabditis elegans reveals a gut-to-muscle stress signaling pathway that activates Hsp70 expression in muscle cells independently of HSF-1, enhancing both stress resistance and longevity.

## Introduction

The preservation of protein homeostasis (proteostasis) is central for the maintenance of cellular and organismal health during environmental and physiological challenges. In multicellular organisms, intercellular signaling processes are essential for organismal development, differentiation, and cell growth [1], as well as for the maintenance of organismal proteostasis [2–5]. The cell nonautonomous regulation of the HSR, the unfolded protein response of the endoplasmic reticulum (UPR^ER^), and the mitochondria (UPR^mito^) [4–8] is playing a key role in the coordination of proteostasis across tissues.

The nervous system has a unique role in this process as it integrates neuronal stimuli for the transmission of a stress response to a distal tissue. It achieves this through wired synaptic connections whereby neurotransmitters such as serotonin and tyramine function as key regulators of the cell nonautonomous HSR or the cell nonautonomous UPR^ER^, respectively [4,9–14]. Non-wired neuronal connections such as neuropeptides and Wnt signaling are also involved in the cell nonautonomous regulation of the HSR, the UPR^ER^, and the UPR^mito^ [7,15]. In addition to the nervous system, the *Caenorhabditis elegans* gut, being a major secretory organ, is another key tissue central for the regulation of organismal proteostasis. It achieves this via the release of neuropeptides, metabolites [16–18], innate immune peptides [19–22], as well as via lysosomal signaling [11]. However, we do not know the identity of specific signaling cues activated by the gut that result in the up-regulation of proteostasis regulators, such as chaperones in different tissues.

We have previously identified transcellular chaperone signaling (TCS) as a cell-nonautonomous stress response mechanism that mediates the activation of protective chaperone expression from one tissue to another [2,21]. TCS is induced by altering the expression levels of the molecular chaperone Hsp90 in specific tissues [2]. For example, neuron- or gut-specific overexpression of Hsp90 leads to a compensatory up-regulation of the same chaperone in muscle cells [2] that safeguards against chronic stresses such as age-associated amyloid protein misfolding [21]. This is achieved via the activation of the transcription factor PQM-1 in the neurons and the intestine that up-regulates extracellular innate immune peptides such as *clec-41* to coordinate organismal proteostasis via TCS [21]. Conversely, reducing Hsp90 expression in the nervous system or the gut leads to the cell nonautonomous up-regulation of *hsp-70* (*C*. *elegans* Hsp72/HSPA1A) that protects *C*. *elegans* from heat stress [2].

Hsp90 is involved in the negative regulation of heat shock factor 1 (HSF-1) and the cytosolic heat shock response (HSR) [23,24]. Being part of a multichaperone complex, Hsp90 is involved in sequestering HSF-1 monomers in the absence of stress and contributes to the deceleration of HSF-1 activity after a sufficient amount of heat shock proteins have been induced following stress [23,25]. Hsp90 inhibition leads to HSF-1 activation and results in the up-regulation of heat shock proteins, including heat-inducible Hsp70 [26–29]. However, it is not clear whether this or a similar mechanism is induced upon tissue-specific Hsp90 knockdown that regulates transcellular activation of HSF-1 and *hsp-70* induction across tissues in *C*. *elegans*.

Here, we examined how knockdown of *hsp-90* in the *C*. *elegans* intestine induces TCS-mediated expression of *hsp-70* in muscle cells. Using a combinatorial approach by analyzing gene expression profiles and a forward genetic screen, we identified *txt* genes and the homeodomain transcription factor CEH-58 as important mediators for TCS between the gut and the muscle. Surprisingly, TCS-mediated induction of *hsp-70* in muscle cells is suppressed by HSF-1 and requires the transcription factor CEH-58. Conversely, CEH-58 suppresses the HSF-1–mediated HSR. This antagonistic regulatory relationship between both transcription factors ensures only 1 type of organismal stress response is induced to mediate heat stress survival in *C*. *elegans*. Our data shows that TCS is an organismal stress response distinct from the canonical HSF-1–mediated HSR that activates *hsp-70* expression in an HSF-1–independent manner.

## Results

### Intestine-specific knockdown of *hsp-90* induces TCS-mediated *hsp-70* expression and extends lifespan

**Fig 1:**
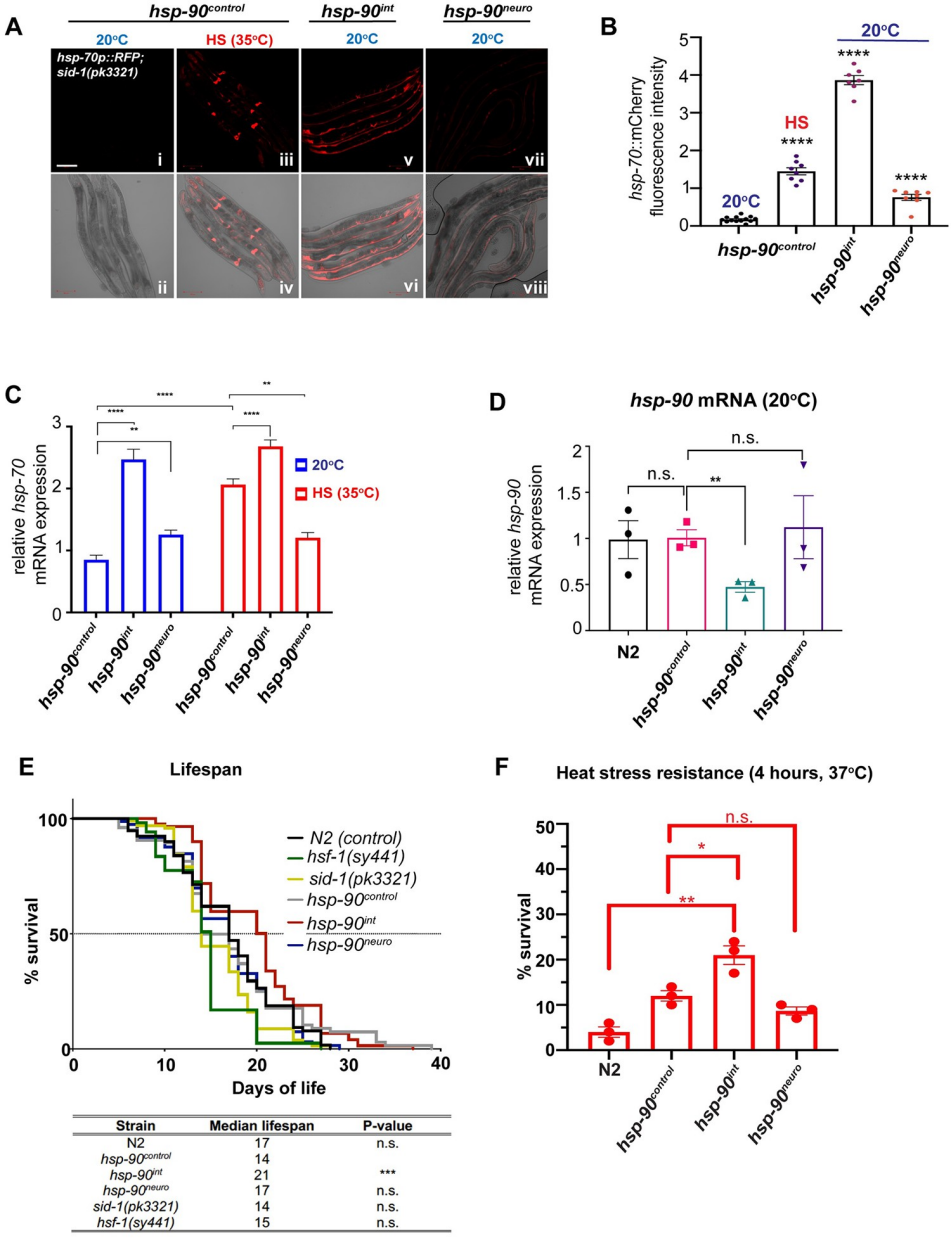
TCS induces *hsp-70* cell nonautonomously in muscle cells and increases lifespan and stress resistance. (**A**) HS (1 h; 35°C) induces expression of the *hsp-70p*::*mCherry* reporter in the spermatheca and pharynx (**iii, iv**). Intestine- (**v, vi**) and neuron-specific (***vii*, *viii***) *hsp-90* knockdown induces *hsp-70p*::*mCherry* expression in muscle cells at 20°C. (**B**) Quantification of *hsp-70p*::*mCherry* fluorescence intensity induced by TCS in *hsp-90*^*int*^ and *hsp-90*^*neuro*^ compared to HS-induced *hsp-70p*::*mCherry* expression in control animals (*hsp-90*^*control*^). At least 5 animals per image and 3 biological replicates. Significance compared to mean fluorescence intensity in *hsp-90*^control^ was determined using one-way ANOVA. (**C**) Whole-animal *hsp-70* mRNA levels of *hsp-90*^*int*^ and *hsp-90*^*neuro*^ animals compared to *hsp-90*^control^ nematodes at 20°C and after a 1-h HS at 35°C. (**D**) Whole-animal *hsp-90* mRNA levels in *hsp-90*^*int*^ and *hsp-90*^*neuro*^ animals compared to *hsp-90*^control^. (**C, D**) Bar graphs represent the average of 3 biological replicates of 50 animals per RNAi and/or temperature condition. Error bars represent SEM of the 3 biological replicates. The statistical significance was determined using (**C**) two-way ANOVA and (**D**) Student’s *t* test, relative to the control strain *hsp-90*^control^. (**E**) Lifespan of *hsp-90*^*int*^ and *hsp-90*^*neuro*^ compared to *hsp-90*^control^ and N2 animals. *hsf-1(sy441)* and *sid-1(pk3321)* strains were used as controls. *n* = 100 animals per strain. Survival curves were compared using Grehan–Breslow–Wilcoxon test. (**F**) Thermotolerance following a 2- and 4-h heat shock at 37°C. *n* > 3 replicates of 50 animals per strain per time point. Significance compared to *hsp-90*^control^ was determined using Student’s *t* test. (**B–F**) *P < 0.05; **P < 0.01; ***P < 0.001; ****P < 0.0001; n.s. = not significant. Source data underlying Fig 1B–F are provided in S1 Data. HSF-1, heat shock factor 1; RNAi, RNA interference; TCS, transcellular chaperone signaling.

We have previously shown that tissue-specific knockdown of *hsp-90* in the gut and neurons results in the cell-nonautonomous up-regulation of *hsp-70* in multiple tissues of *C*. *elegans*, a process we termed transcellular chaperone signaling (TCS) [2]. To further investigate how *hsp-70* expression is activated from one tissue to another, we used *C*. *elegans* strains expressing either an intestine-specific (*hsp-90*^*int*^*) or neuron-specific* (*hsp-90*^*neuro*^) hairpin RNA interference (RNAi) construct against *hsp-90* [2,30]. Both strains show a particular induction of the heat-inducible *hsp-70p*::*mCherry* reporter in the body wall muscle at the permissive temperature (20°C), corresponding to a 22-fold induction of *hsp-70p*::*mCherry* fluorescence intensity in the *hsp-90*^*int*^ strain and a 4-fold induction in the *hsp-90*^*neuro*^ strain (Fig 1A and 1B). The *hsp-70p*::*mCherry* reporter is heat-inducible; therefore, no mCherry fluorescence is detected at 20°C in control (*hsp-90*^*control*^) animals that contain the same genetic background as *hsp-90*^*int*^ and *hsp-90*^*neuro*^ strains but lack the *hp-RNAi* construct (Fig 1A). A 1-h HS (35°C) induces the *hsp-70p*::*mCherry* reporter 8-fold in *hsp-90*^*control*^ animals (Fig 1B), primarily in spermatheca, intestine, and pharynx as reported previously (Fig 1A) [31]. This highlights that the *hsp-70* tissue expression pattern induced by external HS is different from that induced by tissue-specific *hsp-90* knockdown. We confirmed induction of *hsp-70* expression and knockdown of *hsp-90* expression by measuring endogenous transcript levels using quantitative real-time PCR. While knockdown of *hsp-90* in the intestine reduced whole-animal *hsp-90* mRNA levels by 50% (Fig 1D), endogenous *hsp-70* transcripts were induced 2.5-fold compared to *hsp-90*^*control*^ animals at 20°C but did not further increase upon HS in the *hsp-90*^*int*^ strain **(**Fig 1C; ***P* < 0.0001)**. In the *hsp-90*^*neuro*^ strain *hsp-70* mRNA levels were induced 1.2-fold at 20°C and also did not further increase upon HS **(**Fig 1C; ***P* < 0.01)**. Despite a detectable induction of *hsp-70*, *hsp-90* transcripts were not measurably reduced in this strain (Fig 1D). Importantly, gut-specific *hsp-90* RNAi does not result in developmental delays compared to the control (*hsp-90*^*control*^), whereas *hsp-90*^*neuro*^ is developmentally delayed (S1A Fig). The egg laying rate and viability of hatched progeny is unaffected in *hsp-90*^*int*^, but reduced in *hsp-90*^*neuro*^ (S1B and S1C Fig). Likewise, myofilaments examined by visualizing the subcellular localization of myosin heavy chain A (*myo-3*) using a *myo-3p*::*GFP* reporter strain was unaffected in day 1 *hsp-90*^*int*^ adult animals, indicating no developmental alterations for muscle development (S1D Fig).

Overall, these results demonstrate that tissue-specific *hsp-90* hairpin RNAi leads to constitutive up-regulation of *hsp-70* at the permissive temperature, particularly in body wall muscle cells.

To examine whether constitutive induction of *hsp-70* in *hsp-90*^*int*^ and *hsp-90*^*neuro*^ benefits *C*. *elegans* at the organismal level, we measured the effects on lifespan and resistance to heat stress. *hsp-90*^*int*^ animals showed a 50% increase in median lifespan (21 days; *P* < 0.0001) compared to *hsp-90*^*control*^ animals (14 days median lifespan; *P* < 0.0001), whereas no effect was measured in *hsp-90*^*neuro*^ animals (Fig 1E). By comparison, a *hsf-1(sy441)* hypomorph mutant that is deficient in its ability to induce heat shock proteins effectively shows a reduced median lifespan of 15 days, similar to *hsp-90*^*control*^ animals. This indicates that the *sid-1(pk3321)* mutation present in *hsp-90*^*control*^ affects lifespan (Fig 1E).

Exposure to heat stress (4 h, 37°C) detrimentally reduced survival of wild-type *C*. *elegans* (N2 Bristol) and *hsp-90*^*control*^ to 4% and 10%, respectively (Fig 1F). Interestingly, the thermotolerance of *hsp-90*^*int*^ animals was doubled (20%; *P* < 0.05) compared to *hsp-90*^*control*^ animals, whereas *hsp-90*^*neuro*^ animals did not show an increased survival profile, perhaps mirroring the lower level of TCS-mediated *hsp-70* induction (Fig 1F). Indeed, the enhanced stress resistance of *hsp-90*^*int*^ is abolished upon muscle-specific *hsp-70* RNAi after a 4-h HS at 37°C, indicating that increased *hsp-70* levels are crucial for survival (S1E Fig). Overall, this showed that gut-to-muscle-mediated *hsp-70* induction was important to protect against the detrimental consequences of acute heat stress and leads to lifespan extension in *hsp-90*^*int*^.

### TCS-mediated *hsp-70* induction is suppressed by HSF-1

**Fig 2:**
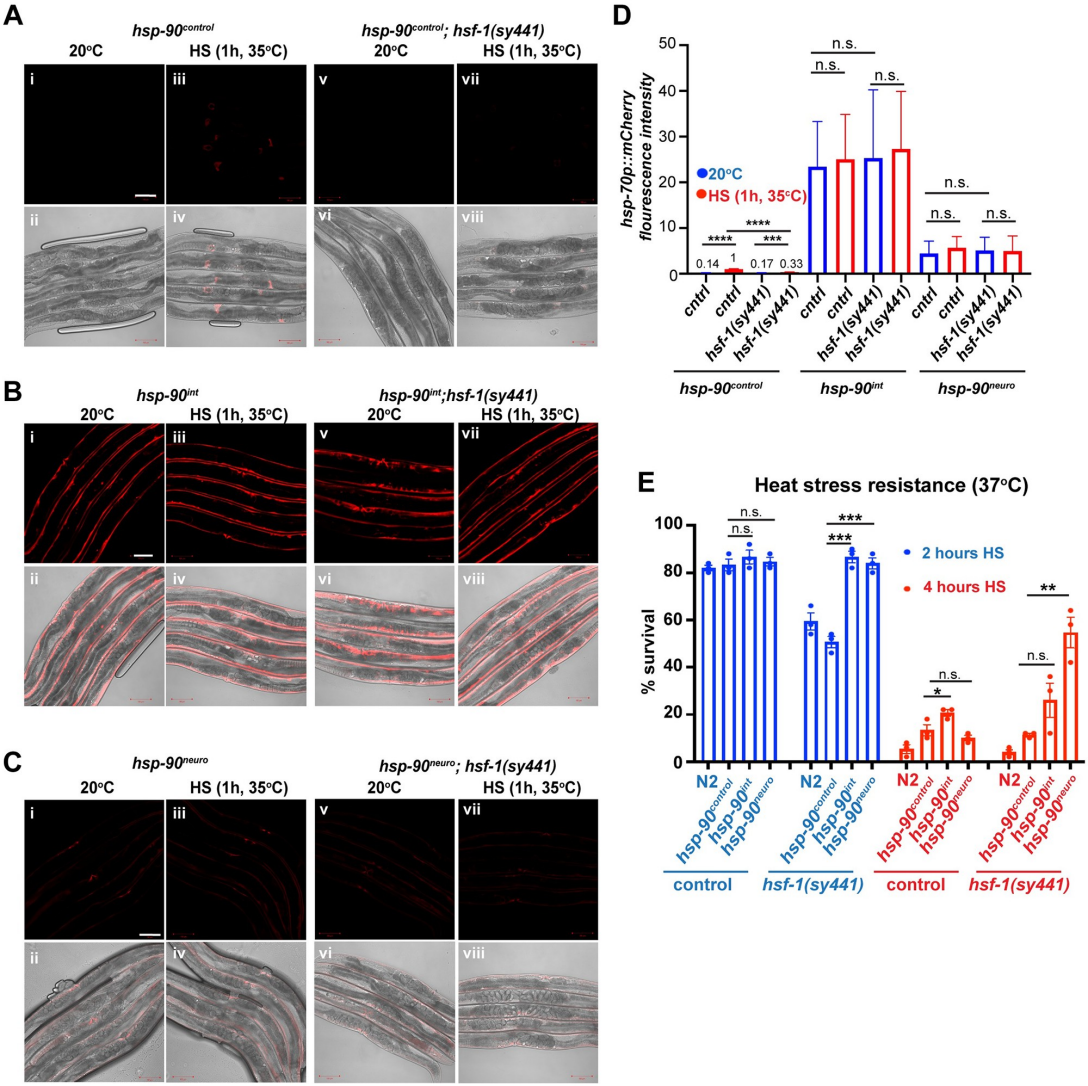
TCS-induced *hsp-70* expression is regulated independently of *hsf-1*. (**A**) The *hsp-70p*::*mCherry* reporter is not expressed in *hsp-90*^*control*^ **(i, ii)** and *hsp-90*^*control*^*;hsf-1(sy441)* **(v, vi)** at 20°C. *hsp-70p*::*mCherry* is induced in *hsp-90*^*control*^ after a 1-h HS at 35°C **(iii, iv)**, but not in *hsp-90*^*control*^*;hsf-1(sy441)* mutants **(vii, viii)**. (**B**) Tissue-specific knockdown of *hsp-90* in the intestine (*hsp-90*^*int*^) induces the *hsp-70p*::*mCherry* reporter in the muscle at 20°C **(i, ii)** and after HS at 35°C **(iii, iv)** to a comparable level. *hsp-70p*::*mCherry* expression in *hsp-90*^*int*^ animals before or during HS (1-h at 35°C) is independent of *hsf-1* (**v–viii**). (**C**) Expression of the *hsp-70p*::*mCherry* reporter in *hsp-90*^*neuro*^ **(i, ii; v, vi)** and *hsp-90*^*int*^;*hsf-1(sy441)* animals **(iii, iv; vii, viii)** before and after HS. (**D**) Quantification of *hsp-70p*::*mCherry* fluorescence intensity at 20°C or after HS (35°C) in control (*hsp-90*^*control*^) or *hsp-90*^*int*^ and *hsp-90*^*neu*^ animals harboring wild-type or mutated *hsf-1*. (**E**) Survival of *hsp-90*^*int*^*;hsf-1(sy441)* and *hsp-90*^*neu*^;*hsf-1(sy441)* animals compared to *hsp-90*^control^ after a 2- and 4-h HS at 37°C. N2 nematodes were used as an additional control. (**D, E**) Bar graphs represent the average of 3 biological replicates of 50 animals per condition. Error bars represent SEM of the 3 biological replicates. Significance compared to *hsp-90*^*control*^ was determined using Student’s *t* test. *P < 0.05; **P < 0.01; ***P < 0.001; ****P < 0.0001; n.s. = not significant. Source data for Fig 2D and 2E is provided in S2 Data. HSF-1, heat shock factor 1; TCS, transcellular chaperone signaling.

HSF-1 regulates the cytosolic HSR and is required for the up-regulation of heat-inducible *hsp-70* after HS or *hsp-90* knockdown [23,25,27–29]. We therefore examined whether intestine- or neuron-specific *hsp-90 hp-RNAi* depended on functional HSF-1 to induce *hsp-70* in muscle tissue and increase organismal survival. To investigate this, we crossed *hsf-1(sy441)* mutants that cannot induce a proper HSR [32] into the genetic background of *hsp-90*^*control*^, *hsp-90*^*int*^, and *hsp-90*^*neuro*^ animals expressing the heat-inducible *hsp-70p*::*mCherry* promoter. As expected, a 1-h HS treatment at 35°C induced *hsp-70p*::*mCherry* fluorescence 10-fold in control (*hsp-90*^*control*^*)* animals (Fig 2D; ***P* < 0.0001**), but was reduced by >50% in *hsp-90*^*control*^*;hsf-1(sy441)* animals (Fig 2A and 2D; ***P* < 0.001**). Consistent with this observation, survival rates of *hsf-1(sy441)* and *hsp-90*^*control*^*;hsf-1(sy441)* animals decreased to 55% after a 2-h exposure to 37°C and further dropped to below 10% after 4 h of HS (Fig 2E). Unexpectedly, the *hsf-1(sy441)* allele rendering HSF-1 dysfunctional had no effect on *hsp-70* reporter expression in *hsp-90*^*int*^ and *hsp-90*^*neuro*^ animals at 20°C or after HS treatment, with *hsp-70p*::*mCherry* fluorescence intensity remaining constant during both conditions (Fig 2B–2D). Moreover, *hsp-90*^*int*^*;hsf-1(sy441)* and *hsp-90*^*neuro*^*;hsf-1(sy441)* animals stably endured 2- and 4-h of heat stress exposure at 37°C, with 26% (n.s.) of *hsp-90*^*int*^*;hsf-1(sy441) and 55%* (*P* < 0.01) *of hsp-90*^*neuro*^*;hsf-1(sy441)* animals surviving 4-h at 37°C (Fig 2E). This result shows that TCS-mediated *hsp-70* up-regulation enhances heat stress resistance and suggests that *hsf-1* acts as a suppressor of TCS. This indicates that TCS may be regulated by a molecular mechanism distinct from the canonical HSF-1–mediated HSR, i.e., HSF-1–dependent gene expression upon heat shock.

### TCS is fundamentally different from the HSF-1–mediated HSR

**Fig 3:**
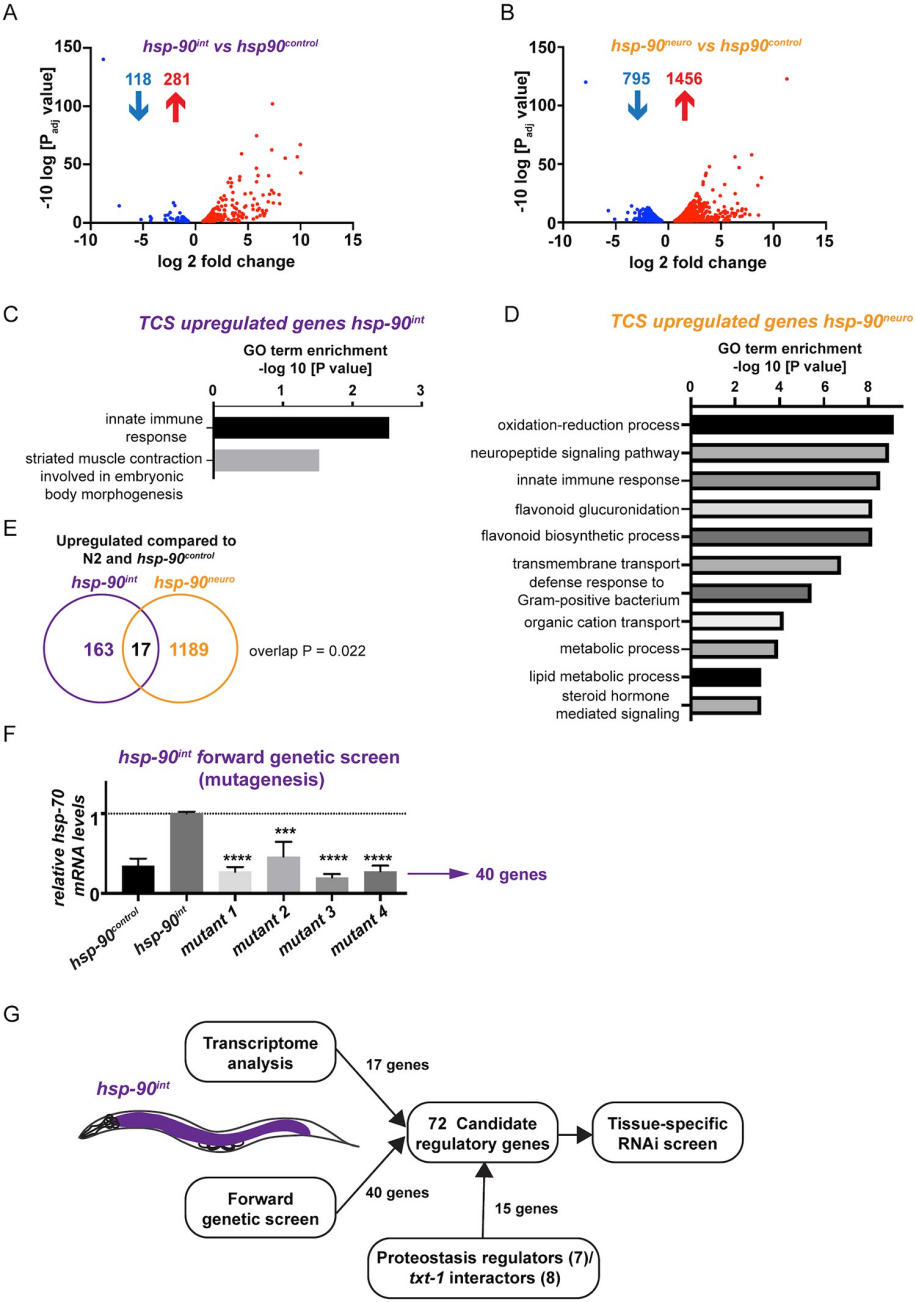
Transcriptional profiling (RNA-Seq) and a forward genetic mutagenesis screen identify candidate genes required for TCS. (**A**) RNA-Seq scatterplot showing log 2-fold change in expression levels of genes (*P* < 0.05) differentially regulated (118 down-regulated; 281 up-regulated) in the TCS-activated strain *hsp-90*^*int*^ compared to *hsp-90*^*control*^. (**B**) RNA-Seq scatterplot showing log 2-fold change in expression levels of genes (*P* < 0.05) differentially regulated (795 down-regulated; 1,456 up-regulated) in the TCS-activated strain *hsp-90*^*neuro*^ compared to *hsp-90*^*control*^. (**C**) GO enrichment analysis of genes up-regulated by TCS in *hsp-90*^*int*^. GO categories are reported as the -log 10 transformation of the *P* value. (**D**) GO enrichment analysis of genes up-regulated by TCS in *hsp-90*^*neuro*^. (**E**) Venn diagram analysis of genes up-regulated in both *hsp-90*^*int*^ and *hsp-90*^*neu*^. *P* value overlap = 0.022 was calculated using probability mass function of overlap size based on hypergeometric distribution. (**F**) *hsp-90*^*int*^ mutants isolated after an EMS mutagenesis screen showing reduced global *hsp-70* mRNA expression that are comparable to *hsp-90*^*control*^ control animals. WGS analysis identified 40 mutated genes in the isolated *hsp-90*^*int*^ mutants. See also S2 Fig. Bar graphs represent the average of 3 biological replicates of >50 animals per strain; error bars represent SEM of the 3 biological replicates. Significance compared to the original strain *hsp-90*^*int*^ (pre-mutagenesis) was determined using Student’s *t* test. ***P < 0.001; ****P < 0.0001. (**G**) Flowchart of identification of the 72 candidate genes resulting from the RNA-Seq analysis (17 genes), the mutagenesis and WGS analysis (40 genes), and proteostasis regulators and *txt-1* interactors (15 genes) that were taken further for a tissue-specific RNAi screen. Note that only 13 (out of 17) candidates of the RNA-Seq analysis (S1 Table) and 31 candidates (out of 40) of the WGS analysis (S2 Table) could be used for further RNAi analysis, as some candidate genes were not present in the genome-wide Ahringer RNAi library. Source data for Fig 3A–D and 3F is provided in S3 Data. GO, Gene Ontology; RNAi, RNA interference; TCS, transcellular chaperone signaling; WGS, whole-genome sequencing.

To investigate how TCS differs from the HSF-1–mediated HSR, we first analyzed the transcriptional expression profile in *hsp-90*^*int*^ and *hsp-90*^*neuro*^ animals compared to the *hsp-90*^*control*^ strain using RNA-Seq. In *hsp-90*^*int*^ worms, 281 genes were up-regulated and 118 genes were down-regulated at the permissive temperature (20°C) (Fig 3A), whereas *hsp-90*^*neuro*^ animals revealed a larger group of 1,456 genes being up-regulated and 795 genes down-regulated (Fig 3B). The 3 most enriched Gene Ontology (GO) terms in *hsp-90*^*neuro*^ animals related to genes involved in neuropeptide signaling, the innate immune response, and transmembrane transport, suggesting a potential involvement in intercellular signaling processes (Fig 3D). *hsp-90*^*int*^ animals showed a clear enrichment for genes involved in the innate immune response and striated muscle contraction involved in embryonic body morphogenesis, which could be reflective of the strong *hsp-70p*::*mCherry* up-regulation in the muscle in these animals (Figs 1A and 3C), albeit muscle development being unaffected (S1D Fig).

Both “TCS-activated” *hsp-90*^*int*^ and *hsp-90*^*neuro*^ strains (Fig 3E and S1 Table) share a set of 17 up-regulated genes enriched for cell membrane proteins and extracellular soluble proteins, thus indicating a role for intercellular signaling processes that could regulate TCS (S1 Table). Interestingly, only few chaperones of the Hsp70 family were up-regulated in *hsp-90*^*int*^ animals (*F44E5*.*4*, *F44E5*.*5*, and *hsp-70*), while no chaperone genes were down-regulated (S2 Fig). Likewise, few chaperones were induced in *hsp-90*^*neuro*^ animals including small heat shock proteins (*hsp-12*.*3* and *hsp-12*.*6*) and 4 cadmium responsive ER chaperones (*cdr-2*, *cdr-4*, *cdr-5*, and *cdr-6*) (S2 Fig). By comparison, none of the TCS up-regulated gene datasets were identified during HS conditions. Following HS, the most enriched GO terms of HSF-1 up-regulated genes are related to cuticle structure, translation and response to stress including members of the HSP16 (alphaB-crystallin) family of heat shock proteins [33]. Comparison of the 815 genes induced by HS in wild-type *C*. *elegans* [33] with our TCS dataset (*hsp-90*^*int*^) showed only a 1.7% overlap between the HSF-1–mediated HSR and TCS **(**S1B Fig; ***P* < 0.04)**. Thus, the transcriptional program induced by tissue-specific knockdown of *hsp-90*, which activates TCS, is fundamentally different from the “canonical” HSF-1–mediated HSR that is triggered by external HS.

### Identification of candidate genes regulating TCS

In addition to RNA-Seq profiling, we undertook a forward genetic (mutagenesis) screen of *hsp-90*^*int*^ animals to identify genes underlying the transcellular up-regulation of *hsp-70* from the intestine to the muscle. We decided to focus on the effects of gut-to-muscle–mediated TCS (*hsp-90*^*int*^ strain) for the mutagenesis screen, because of its strong *hsp-70p*::*mCherry* reporter expression in the body wall muscle (Fig 1A), which allowed for visual detection of increased or reduced mCherry fluorescence intensity. Four mutant strains were isolated showing reduced *hsp-70* reporter expression compared to the original *hsp-90*^*int*^ strain, indicating that these strains harbored a mutation in one or more genes required for TCS-mediated *hsp-70* up-regulation (Fig 3F, S3A and S3B Fig). To map the potential phenotype-causing mutations, we performed whole-genome sequencing (WGS) combined with a SNP-based mapping step [34] leading to the identification of 40 candidate genes (corresponding to 45 SNPs) that could potentially underlie reduced TCS (Fig 3F and S2 Table). Both WGS analysis and measurement of endogenous (whole-animal) *hsp-70* transcripts by qRT-PCR confirmed that the reduced *hsp-70p*::*mCherry* expression in these mutants was not due to a mutation in the promoter region of the *hsp-70p*::*mCherry* reporter (Fig 3F and S2 Table).

**Fig 4:**
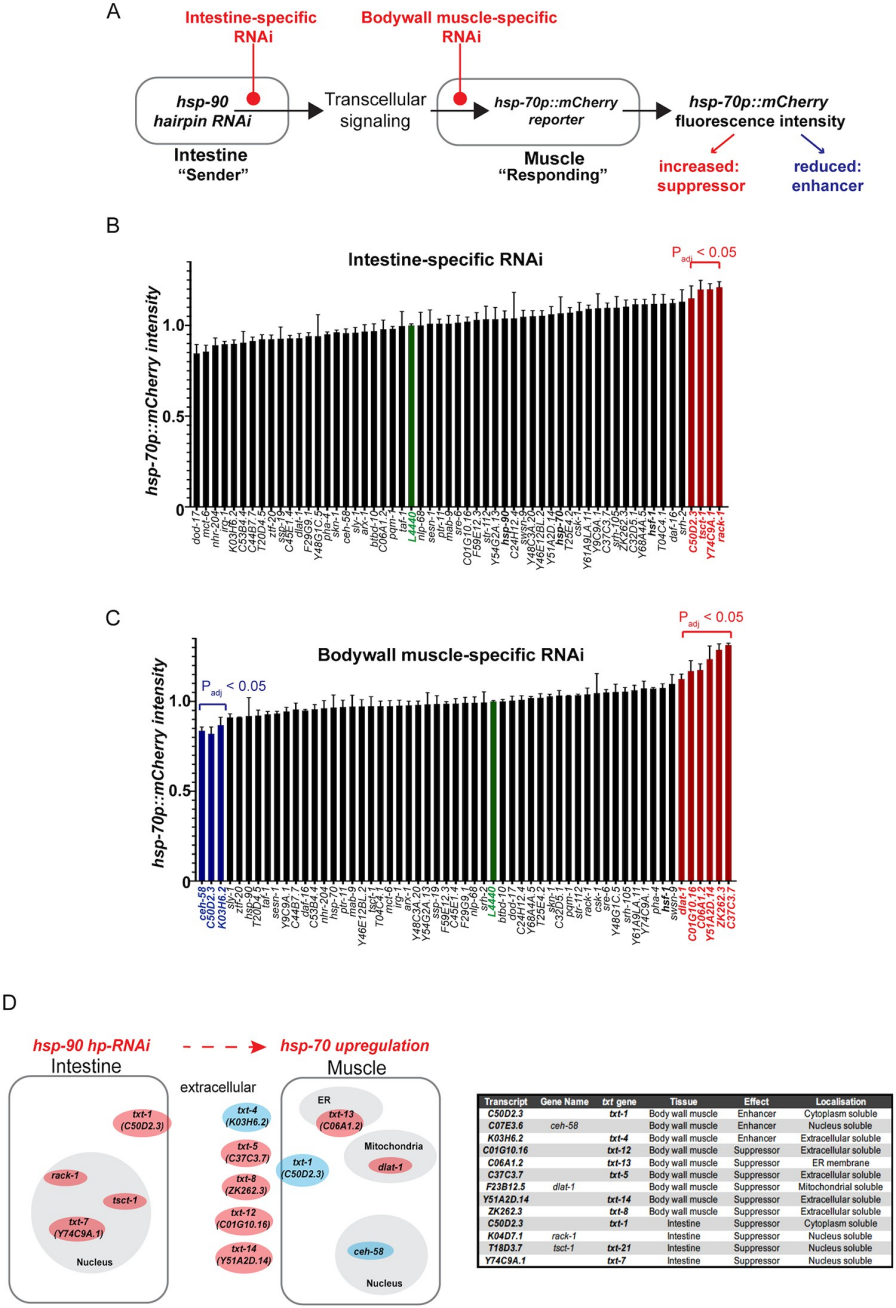
*txt* genes are required for TCS-mediated *hsp-70* induction from intestine-to-muscle. (**A**) Schematic of the intestine- and muscle-specific RNAi screen using candidate genes that are either knocked down in the intestine, which expresses an *hsp-90 hp-RNAi* construct (sender tissue) or in the muscle where *hsp-70p*::*mCherry* fluorescence intensity is induced by TCS (“receiving tissue”). Differences in the level of TCS-induced *hsp-70p*::*mCherry* fluorescence intensity after candidate gene RNAi will point to regulators (enhancers or suppressors) of TCS. (**B**) Intestine-specific candidate RNAi screen using *hsp-70p*::*mCherry* fluorescence intensity as a read-out. Red bars denote tissue-specific RNAi hits that result in a significantly increased fluorescence respectively (P_adj_; adjusted *P* value < 0.05). (**C**) Muscle-specific RNAi screen using *hsp-70p*::*mCherry* fluorescence intensity as a read-out. Blue and red bars denote tissue-specific RNAi causing significantly reduced or increased fluorescence, respectively (adjusted *p* value < 0.05). (**B and C**) Bar graphs represent the average of 3 biological replicates of 50 animals per condition. Error bars represent SEM of the 3 biological replicates. Significance compared to control RNAi (L4440, green column) determined by one-way ANOVA with correction for multiple testing using the two-stage linear step-up procedure of Benjamini, Krieger, and Yekutieli. Source data for Fig 4B and 4C is provided in S4 Data. (**D**) Diagram and table of predicted subcellular localizations of proteins encoded by *txt* genes, in the relevant tissues. Blue denotes fluorescence enhancers, red denotes suppressors. The 5 genes shown outside the body wall muscle encode predicted extracellular soluble proteins. *txt-1 (C50D2*.*3)* expressed in the cytosol was identified as an intestine-specific suppressor and as a muscle-specific enhancer of TCS-mediated *hsp-70* induction in the muscle. RNAi, RNA interference; TCS, transcellular chaperone signaling.

Together, the RNA-seq profiling identified 17 candidate genes (S1 Table) and the forward genetic screen 40 candidate mutations (S2 Table) that could potentially regulate the TCS-mediated *hsp-70* activation from the intestine to the muscle. We named these candidate genes “*txt*” genes, “*T**CS-cross(**X**)-**T**issue*” (Fig 3G and S1 and S2 Tables). To identify which candidate genes underlie the reduced TCS phenotype, we performed tissue-specific RNAi screens. Because the *hsp-90*^*int*^ strain is RNAi resistant, due to the *sid-1(pk3321)* mutant background, we decided to perform these tissue-specific RNAi screens in strains that are sensitive to RNAi in either muscle (strain PVH171) or intestinal tissues (strain PVH172) (S3 Table). This allowed us to determine in which tissue the candidate genes (S1 and S2 Tables) act to facilitate TCS from the stress-perceiving “sender tissue” (intestine) to the *hsp-70p*::*mCherry* inducing and responding muscle tissue (**see** Figs 3G and 4A **for a flowchart**).

### *txt* genes are required for TCS-mediated *hsp-70* induction from intestine-to-muscle

Out of the 17 candidates of the RNA-Seq analysis, 13 genes were screened (S1 Table) and out of the 40 candidates identified through the WGS analysis (S2 Table), 31 genes were screened for tissue-specific RNAi analysis. Less candidate genes were screened because not all RNAi clones were present in the genome-wide Ahringer RNAi library. In addition to the *txt* genes that were identified from the RNA-Seq and WGS analysis, we also included 15 genes known to be involved in the regulation of proteostasis including *pha-4*, *skn-1*, *pqm-1*, and *daf-16* [2,21,35–37], as well as previously identified interaction partners of *txt-1/C50D2*.*3* (8 genes) [38] as additional candidates for the tissue-specific RNAi screen (S4 Table). We utilized *hsp-90*^*int*^ strains that allowed for muscle-specific (strain PVH171) or intestine-specific (strain PVH172) RNAi-mediated knockdown of the 72 candidate genes and measured *hsp-70p*::*mCherry* fluorescence intensity as a read-out [30]. Note that whole-animal RNAi cannot be used in *hsp-90*^*int*^ due to the *sid-1(pk3321)* mutant background that renders *C*. *elegans* insensitive to RNAi at the systemic level [30,39,40].

The intestine-specific RNAi screen identified 4 genes (*C50D2*.*3; tsct-1; Y74C9A*.*1* and *rack-1*) as intestine-specific suppressors of TCS-mediated *hsp-70p*::*mCherry* induction in the muscle **(**Fig 4B; ***P* < 0.05)**. The body wall muscle-specific RNAi screen identified 3 genes acting as muscle-specific enhancers (*C50D2*.*3; ceh-58; K03H6*.*2*) and 6 genes acting as suppressors (*dlat-1; C01G10*.*16; Y51A2D*.*14; ZK262*.*3; C37C3*.*7; C06A1*.*2*) of TCS-mediated *hsp-70* expression in the muscle **(**Fig 4C; ***P* < 0.05)**. Fig 4D summarizes the predicted subcellular localization of these gene hits (using the DeepLoc 1.0 web tool) and the tissue in which they act as enhancers or suppressors (Fig 4D).

Among the gene hits, *txt-1 (C50D2*.*3)*, raised specific interest as it appeared to function as a muscle-specific enhancer and an intestine-specific suppressor of TCS-mediated *hsp-70* expression (Fig 4B and 4C), suggesting a key role for *txt-1* in TCS. Interestingly, *txt-1* encodes for a predicted PDZ domain protein [38], which often act as scaffolds for larger multiprotein complexes at the inner cell membrane and are involved in transmembrane receptor organization and vesicle trafficking [41,42]. Such a function could be particularly relevant for intercellular signaling with *txt-1* acting as a key node at the plasma membrane of muscle cells that receives the intercellular signal to induce *hsp-70* expression. Moreover, the transcription factor *ceh-58*, a direct and known interactor of *txt-1* [38] was also identified as an enhancer of TCS-induced *hsp-70* expression in the muscle (Fig 4C) and encodes a homeobox transcription factor. Indeed, a CEH-58 consensus motif (TAATTA/G) is present in the promoter of the *hsp-70* gene 800 base pairs upstream of the *hsp-70* transcription start site ([43]; CIS-BP database; S4A Fig). This suggested that *txt-1* and *ceh-58* could indeed be components of an inter-tissue signaling cue that transmits a TCS signal from the intestine to induce *hsp-70* expression in the muscle.

To understand whether other chaperones that are differentially regulated in *hsp-90*^*int*^ could also be regulated by CEH-58, we scanned all 399 differentially regulated genes in this strain for the occurrence of both consensus motifs, i.e., CEH-58 (TAATTA/G) and HSF-1 (TTCYAGAA) motifs. The analysis yielded 34 gene promoters containing at least 1 CEH-58 motif (S5 Table and S4C Fig) and 125 gene promoters containing at least 1 HSF-1 motif (S5 Table and S4E Fig). Four of the differentially expressed genes in *hsp-90*^*int*^ are chaperones, of which only the promoter of *hsp-70* had 1 CEH-58 motif and 3 HSF-1 motifs (S5 Table). A total of 12 genes differentially regulated in *hsp-90*^*int*^ contained at least 1 motif for both CEH-58 and HSF1 (S4B and S4D Fig). GO term analysis showed an enrichment for genes involved in the unfolded protein response, signal transduction, and membrane proteins (S4D Fig). Although the only chaperone regulated by both transcription factors is *hsp-70* in the *hsp-90*^*int*^ strain, other proteostasis-related genes can be regulated by HSF-1 and CEH-58. Thus, both transcription factors may be involved in the regulation of organismal proteostasis.

### Activation of TCS acts as a “switch” that shifts control of *hsp-70* expression from HSF-1 to TXT-1/CEH-58

**Fig 5:**
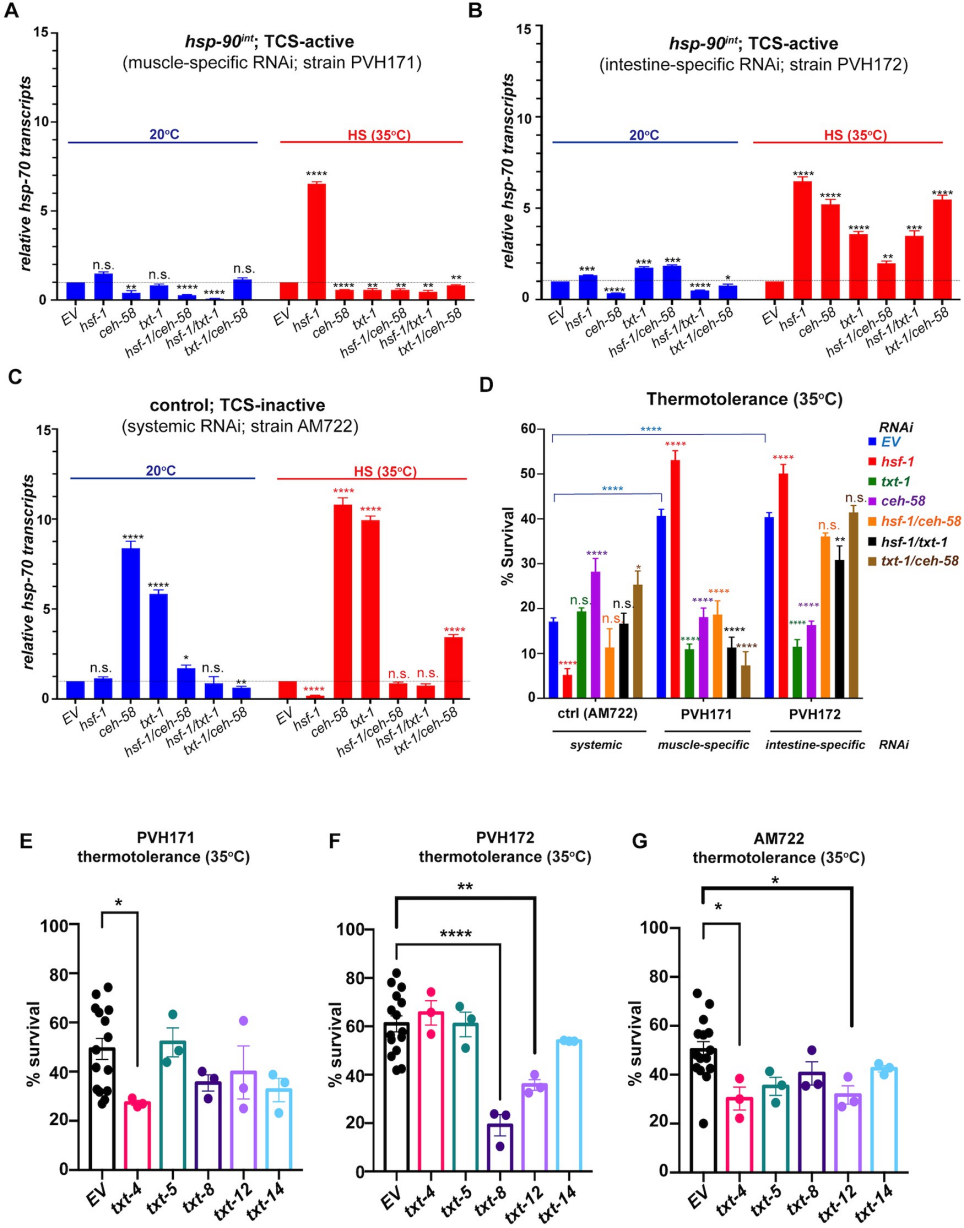
*txt-1* is required for TCS-mediated *hsp-70* induction during HS independent of *hsf-1*. (**A**) TCS-mediated *hsp-70* induction is facilitated by *txt-1* and *ceh-58* in the muscle. Quantification of whole-animal *hsp-70* transcripts in *hsp-90*^*int*^ animals at 20°C and HS (35°C), during muscle-specific RNAi in (strain PVH171) against *hsf-1*, *ceh-58*, *txt-1*, and double RNAi of *hsf-1/ceh-58 hsf-1/txt-1*, *txt-1/ceh-58*. (**B**) TCS-mediated *hsp-70* induction is suppressed by *txt-1* in the intestine. Quantification of whole-animal *hsp-70* transcripts in *hsp-90*^*int*^ animals at 20°C and HS (35°C), during intestine-specific RNAi (strain PVH172) against *hsf-1*, *ceh-58*, *txt-1*, and double RNAi of *hsf-1/ceh-58 hsf-1/txt-1*, *txt-1/ceh-58*. (**C**) *txt-1* and *ceh-58* are suppressors of the HSR. Quantification of whole-animal *hsp-70* transcripts in control animals at 20°C and HS (35°C), allowing for systemic RNAi (strain AM722) against *hsf-1*, *ceh-58*, *txt-1*, and double RNAi of *hsf-1/ceh-58 hsf-1/txt-1*, *txt-1/ceh-58*. (**D**) Thermotolerance after 6-h of heat stress at 35°C of TCS active strains during muscle-specific RNAi (strains PVH171) and intestine-specific RNAi (strain PVH172) compared to a control strain (TCS-inactive; AM722) during systemic RNAi against *hsf-1*, *ceh-58*, *txt-1*, and double RNAi of *hsf-1/ceh-58 hsf-1/txt-1*, *txt-1/ceh-58*. (**A–D**) Bar graphs represent the average of 3 biological replicates of 50 animals per RNAi and/or temperature condition. Error bars represent SEM of the 3 biological replicates. Significance in (**A–C**) was determined using Student’s *t* test. Significance in (**D**) compared to control RNAi (EV) was determined using one-way ANOVA. *P < 0.05; **P < 0.01; ***P < 0.001; ****P < 0.0001; n.s. = not significant. (**E**) Thermotolerance of *hsp-90*^*int*^ during muscle-specific RNAi (strain PVH171) against *txt-4*, *txt-5*, *txt-8*, *txt-12*, and *txt-14* compared to control RNAi (EV). Day 1 adults were exposed to a 6-h heat shock at 35°C. (**F**) Thermotolerance of *hsp-90*^*int*^ during intestine-specific RNAi (strain PVH172) against *txt-4*, *txt-5*, *txt-8*, *txt-12*, and *txt-14* compared to control RNAi (EV). (**G**) Thermotolerance of a control strain (TCS-inactive; AM722) during systemic RNAi against *txt-4*, *txt-5*, *txt-8*, *txt-12*, and *txt-14* compared to control RNAi (EV). (**E–G**) Bar graphs represent the average of 3 biological replicates of 50 animals per RNAi and/or temperature condition. Error bars represent SEM of the 3 biological replicates. Significance compared to control RNAi (EV) was determined using Student’s *t* test. *P < 0.05; **P < 0.01; ***P < 0.001; ****P < 0.0001; n.s. = not significant. Source data for Fig 5A–G is provided in S5 Data. HSR, heat shock response; RNAi, RNA interference; TCS, transcellular chaperone signaling.

To further examine the role of *txt-1* and *ceh-58* in TCS, we investigated how intestine- and muscle-specific RNAi of either gene affected *hsp-70* induction at 20°C and HS and whether this reduced the increased thermotolerance of *hsp-90*^*int*^ animals. Knockdown of *txt-1* or *ceh-58* by RNAi in muscle cells of *hsp-90*^*int*^ not only reduced expression of the *hsp-70p*::*mCherry* in the body wall muscle as identified by the tissue-specific RNAi screen (Fig 4C) but also did not further increase expression of the fluorescent *hsp-70* reporter upon heat shock (S5F Fig). It also resulted in reduced global endogenous *hsp-70* transcripts at 20°C and during HS (Fig 5A), as well as reducing thermotolerance by 50% **(**Fig 5D; ***P* < 0.0001)**. This confirmed that both transcellular signaling components act as facilitators of TCS-induced *hsp-70* expression in muscle cells that is crucial for heat stress survival. Conversely, muscle-specific *hsf-1* RNAi increased *hsp-70* transcripts by 7-fold (Fig 5A) and further improved HS survival rates to >60% compared to a 20% survival rate of control animals (Fig 5D), verifying HSF-1 role’s as a suppressor of TCS. This suppressive function of *hsf-1* is however abolished when knocked down in combination with either *ceh-58* or *txt-1* by RNAi (Fig 5A) suggesting that the interplay between *txt-1* and *hsf-1* or *ceh-58* and *hsf-1* is required for HSF-1’s repressive activity in the muscle (Fig 5A) as well as TCS-mediated survival rates (Fig 5D). The level of *hsf-1*, *txt-1*, or *ceh-58* knockdown in these RNAi experiments was at least 50% (S5H–S5J Fig). Similarly, extracellular peptides *txt-4* and *txt-8* act as facilitators of TCS-mediated *hsp-70* induction in the muscle of *hsp-90*^*int*^ during HS (S5A Fig) and are required for thermotolerance (Fig 5E), indicating that both extracellular peptides could function as transmitters of TCS from the intestine to the muscle, albeit simultaneous *txt-4/txt-8* RNAi had no influence on *hsp-70* expression at 20°C (S5D Fig).

**Fig 6:**
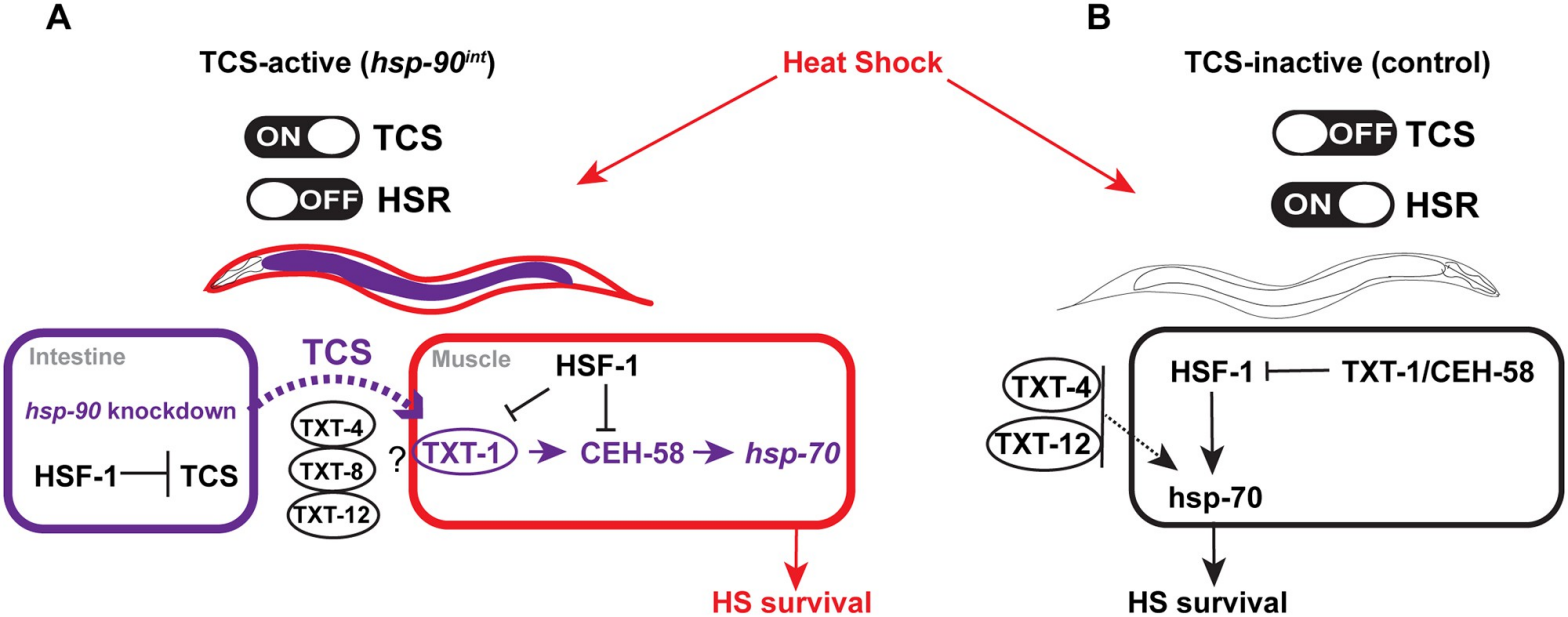
Proposed model explaining the opposing regulatory mechanisms of *hsp-70* induction and survival in TCS-active compared to a TCS-inactive strain. (**A**) Upon *hsp-90* RNAi in the intestine (“TCS-active”) *hsp-70* is induced in the muscle via an *hsf-1*-independent mechanism that depends on TXT-1/CEH-58 signaling in the muscle as well as extracellular peptides TXT-4, TXT-8, and TXT-12. HSF-1 functions as a suppressor of TCS-mediated *hsp-70* expression and survival. (**B**) In control animals (“TCS-inactive”), cell nonautonomous *hsp-70* induction and survival depends on HSF-1 and extracellular peptides TXT-4 and TXT-12 during HS; and is suppressed by TXT-1/CEH-58. HSF-1, heat shock factor 1; TCS, transcellular chaperone signaling.

In a control strain where TCS is not active and RNAi is systemic (strain AM722), *hsf-1* RNAi abolished *hsp-70* expression and thermotolerance as expected (Fig 5C and 5D). Strikingly, the opposite effect is achieved upon *ceh-58* RNAi in the same strain, resulting in a 10-fold induction of global *hsp-70* transcripts even at permissive temperature (Fig 5C) and which also increased heat stress resistance (Fig 5D). Thus, while HSF-1 normally regulates the HSR by inducing *hsp-70* expression and survival in control animals exposed to HS, intestine-specific knockdown of *hsp-90* instead “switches off” the classic HSF-1–mediated HSR. Under these conditions, *hsp-70* expression is dependent on CEH-58 (Fig 6).

The suppressive function of HSF-1 for TCS is also demonstrated in the intestine, as gut-specific *hsf-1* RNAi increased *hsp-70* expression at 20°C and during HS (Fig 5B), as well as further enhancing thermotolerance (Fig 5D). The function of *txt-1* and *ceh-58* in the intestine however differs from their role in the muscle: in the intestine *txt-1* and *ceh-58* both suppress TCS-mediated *hsp-70* expression during HS, albeit *ceh-58* being a facilitator of *hsp-70* expression at 20°C (Fig 5B). Interestingly, heat stress survival rates upon *ceh-58* and *txt-1* RNAi in the intestine are reduced (Fig 5D), despite increased global induction of *hsp-70* transcripts (Fig 5B). Intestine-specific knockdown of extracellular peptides *txt-8* and *txt-12* show a similar picture with reduced survival (Fig 5F) despite increased TCS-mediated *hsp-70* expression (S5B Fig). This suggests that the inhibitory function of *txt-1/ceh-58* and putative extracellular peptides *txt-8* and *txt-12* in the intestine is required for TCS-mediated regulation of survival.

We summarize these findings in a model describing gut-to-muscle stress signaling upon intestine-specific *hsp-90* knockdown and the consequences for heat stress survival (Fig 6). In control strains (“TCS-inactive”), *hsp-70* expression and heat stress survival are regulated in an HSF-1–dependent manner, with putative extracellular peptides TXT-4 and TXT-12 involved in this process. During HS, both TXT-1 and CEH-58 function as suppressors of HSF-1–mediated *hsp-70* induction (Fig 6B).

Knockdown of *hsp-90* in the intestine (“TCS-active strain”) relays a transcellular signal to muscle cells via putative extracellular peptides TXT-4, TXT-8, and TXT-12 (Fig 6A). In the muscle, the membrane-associated TXT-1 protein then facilitates intracellular receipt of the TCS signal towards the transcription factor CEH-58, which promotes *hsp-70* induction and organismal HS survival. TXT-1 may also be required to transduce a signal to HSF-1, as combinatorial knockdown of *txt-1* and *hsf-1* abolishes the suppressive function of HSF-1.

We conclude that TCS and the HSF-1–mediated HSR are regulated in an antagonistic manner, with TCS inhibiting HSF-1 activity, whereas conversely, HSF-1 suppresses TCS-induced TXT-1/CEH-58 signaling (Fig 6).

## Discussion

*Hsp-90* knockdown in the *C*. *elegans* intestine transmits a signal to induce *hsp-70* in muscle cells by utilizing a hitherto uncharacterized signaling pathway. In this work, we identified *txt* genes and the homeodomain transcription factor CEH-58 as TCS mediators involved in the regulation between gut-to-muscle signaling. We find that TCS-mediated *hsp-70* induction relies on CEH-58 in the muscle, rather than HSF-1 which, interestingly, acts as a suppressor of TCS. Vice versa, CEH-58 suppresses *hsp-70* induction and survival following heat stress in TCS-inactive control strains. We propose that TCS and the HSF-1–mediated HSR are regulated in an antagonistic manner that distinguishes between intracellular stress (gut-specific *hsp-90* RNAi) versus external stress (heat shock) to safeguard and ensure organismal heat stress survival.

Inter-tissue signaling can be triggered in the gut to communicate with other tissues, such as during pathogenic infection [19,22,44], or during FOXO-to-FOXO signaling that has a lifespan extending effect via intestinal released lipid signals [18]. This role of the intestine has however received less attention in the context of cellular stress responses and cell nonautonomous induction of molecular chaperones. Our study here investigated such inter-tissue signaling initiated by the gut that results in the up-regulation of *hsp-70* in muscle cells.

Lifespan extending effects and heat stress resistance as a consequence of *hsp-90* inhibition has been previously reported in *C*. *elegans*, by using small molecule drugs such as Radicicol (Monorden) and Tanespimycin [45], and induction of the HSR by systemic *hsp-90* RNAi [46]. However, this beneficial organismal consequence is only achieved if induced after *C*. *elegans* development is completed (at the L4 larval stage). Earlier Hsp90 knockdown at the systemic level otherwise leads to developmental arrest and dauer formation [45,47]. In contrast, in our study intestine-specific *hsp-90* RNAi is induced constitutively from embryonic stages of development onwards, without apparent developmental defects. Previously, systemic *hsp-90* RNAi from L1 stage onwards was shown to induce *hsp-70* in body wall muscle and intestinal cells [31,48] and impacted on gene expression related to oocyte and larval development, collagen and cuticle formation, and intestinal and pharynx development (Eckl and colleagues). However, comparison of gene expression datasets between intestine-specific *hsp-90* RNAi (this study) and systemic *hsp-90* RNAi (Eckl and colleagues) shows only 4 overlapping up-regulated genes. In particular, *hsp-70*, and innate immune genes *clec-76*, *zip-10*, *sdz-35* are up-regulated upon systemic as well as intestine-specific *hsp-90* RNAi. Analysis of muscle integrity and development of *hsp-90*^*int*^ animals shows only a mild developmental delay (S1A Fig) but no impact on muscle structure (S1D Fig). A striking difference is also that small heat shock proteins, including *hsp-16*.*1* and *hsp-16*.*2* are 1 group most up-regulated upon HS [33] and upon systemic *hsp-90* RNAi [46], but not by intestine-specific *hsp-90* RNAi. Noteworthily, although systemic *hsp-90* RNAi leads to transcriptional changes resulting in the impairment of oocyte development [46], our dataset does not reflect any impact on oocyte-related genes. Thus, together this highlights apparent differences between thermal challenges, systemic and tissue-specific knockdown of *hsp-90* and indicates that gene expression changes upon intestine-specific *hsp-90* RNAi does not result in developmental issues.

It is surprising that *hsp-90* knockdown in the intestine does not require HSF-1 to induce *hsp-70* expression in the muscle. Knockdown or inhibition of Hsp90 activity is known to activate HSF-1–mediated expression of heat-inducible Hsp70 [24,25]. HSF-1 has been reported to facilitate intercellular communication between cancer-associated fibroblasts that is required to promote cancer cell growth [49,50]. This is in contrast to our finding in *C*. *elegans*, where HSF-1 functions as a suppressor of intercellular *hsp-70* induction upon gut-specific *hsp-90* knockdown. This suggests that at an organismal level, more complex layers of regulation may be required to achieve the appropriate response in the correct target tissue. Perhaps the choice of an alternative transcription factor such as CEH-58 is necessary to achieve a high level of specificity tailored for a specific cell type. This indicates the requirement for a specific “tissue-code” of organismal proteostasis that ascertains induction of a cell-type–specific stress response in metazoans. The existence of such a tissue code is very likely, given the tissue-specific expression pattern of molecular chaperones in physiological human tissues [51], as well as chaperone networks that are tailored towards specific diseases, including neurodegenerative disorders [52] and cancer [53].

Transcription factors other than HSF-1 are known to be involved in the regulation of molecular chaperones, including FOXO/DAF-16, SKN-1/NRF2, PHA-4, and PQM-1 [21,27,35,54–56]. Although CEH-58 has previously not been implicated in chaperone expression, the *hsp-70* promoter contains a CEH-58 consensus sequence and our data suggests that CEH-58 genetically interacts with HSF-1. Future studies will need to confirm direct binding of CEH-58 to DNA elements in the promoters of stress-responsive heat shock proteins and a potential co-regulation with HSF-1 by direct protein–protein interaction between both transcription factors.

TCS clearly distinguishes itself from an HSF-1–mediated stress response, not only because TCS relies on CEH-58 for heat stress resistance but importantly because the transcriptional profile induced by TCS has little in common with the classic HSR mediated by HSF-1 (S1B Fig). When TCS is induced in the intestine, innate immune response genes are a major up-regulated gene group, rather than HSF-1–dependent heat shock proteins that are among the highest up-regulated genes following external heat stress in *C*. *elegans* [33]. Moreover, TCS induction in the intestine appears to “switch off” the HSF-1–mediated HSR in favor of a TXT-1/CEH-58 mediated signaling route that promotes protective *hsp-70* induction and survival. It is possible that these opposing effects are part of a built-in negative regulatory mechanism that safeguards stress survival.

In addition to CEH-58, TXT-1 is another signaling component, newly identified in this study to respond to intestinal derived TCS signals in muscle cells. TXT-1 is a PDZ (PSD-95, Discs-large, ZO-1) domain containing protein, which serve as signaling scaffolds for efficient and specific signal transduction at defined subcellular sites [42,57]. TXT-1 itself is predicted to be localized at the cell membrane and is expressed in muscle cells and the nervous system in *C*. *elegans*, similar to CEH-58 [58]. Our data shows that TXT-1 and CEH-58 genetically interact as part of an **intra**cellular signaling cue in the *C*. *elegans* body wall muscle, corroborating a previous yeast-two-hybrid study that demonstrated direct interaction between both proteins [38]. Although our study is the first to note an association of TXT-1 with the heat stress response in *C*. *elegans*, its closest human orthologue, DLG5, is a membrane associated guanylate cyclase that is part of the Hippo pathway [59,60] and is involved in the cellular response to heat stress [61].

Extracellular peptides may be important contributors of organismal proteostasis that enable intercellular signaling [62]. Among extracellular peptides identified in our study, TXT-4 is a predicted extracellular lipase expressed in the intestine [58] that is crucial for the response to heat stress even in the absence of TCS. While it is an enhancer of *hsp-70* in muscle cells, it acts as a suppressor in the intestine. This indicates the existence of a negative feedback mechanism between gut and muscle. We speculate that the suppressor role of TXT-1 in the gut is important for this feedback mechanism to allow *hsp-70* induction in the muscle only during gut-induced stress in “TCS-activated animals” (Fig 5A), but not wild type where the opposite occurs (Fig 5C). Another extracellular TCS mediator is TXT-8, a phospholipase implicated in autophagy, which was previously identified as one of the 57 extracellular proteostasis regulators in *C*. *elegans* [20]. Given their potential role in intercellular stress signaling, it will be interesting to follow up on the involvement of these extracellular proteostasis regulators in the regulation of cell nonautonomous stress responses [20]. Interestingly, lipid signals have been previously suggested in FOXO-to-FOXO signaling from the intestine to muscle tissue [18], suggesting a potential wider role for lipases such as TXT-4 and TXT-8 in the regulation of intercellular stress signaling.

Overall, our study highlights that transcellular signaling between the gut and muscle induces an HSF-1–independent stress response in muscle cells. We suggest that the opposing effects between TCS and the HSF-1–mediated HSR are part of a built-in negative regulatory mechanism benefiting organismal survival by monitoring responses in the most effective way.

Indeed, the tissue-specific regulation of the HSR often results in opposing effects that impact organismal proteostasis in *C*. *elegans*, such as the differential activation of DAF-16 and HSF-1–dependent stress responses in distal tissues that is controlled by the nervous system [14], and differences of neuronal signaling responses regulating protein aggregation and acute HS [63]. Further understanding of signaling elements involved in global stress responses that can be initiated in the gut to other tissues will become important for manipulation to promote organismal health. While this study focused on signaling occurring from the gut to the muscle, comparable responses exist in gut-to-brain signaling that could have potential major implications for the treatment of neurodegenerative diseases. For example, the gut-brain axis in mammals plays a crucial role in the pathogenesis of age-associated neurodegenerative maladies, including Alzheimer’s and Parkinson’s disease, as well as amyotrophic lateral sclerosis. This is accomplished through gut microbiota that influence such disease progression profoundly [64,65], as well as the ability of the mammalian brain to sense mechanical, chemical, or bacterially derived stimuli from the gut via gut-released hormones as well as neurotransmitters [66]. In this way, the gut can directly impact organismal proteostasis that is evolutionary conserved. Future research will need to unravel the intercellular signals exchanged between the gut and other organs to fully harness the potential therapeutic interventions arising from this transcellular communication.

## Material and methods

### *C*. *elegans* maintenance and strains

*C*. *elegans* strains used in this study are listed in S3 Table. Worms were maintained at 20°C on NGM agar plates seeded with OP50-1 *E*. *coli* according to standard procedures, unless otherwise stated [67].

### Generation of transgenic strains

Extrachromosomal arrays expressing the neuron- and intestine-specific *hsp-90* hairpin constructs in a *sid-1(pk3321)* mutant background and expressing the *hsp-70p*::*mCherry* reporter (strain AM994) [2] were integrated and backcrossed 5 times with strain AM994, that allows visualization of *hsp-70* induction via the stress-inducible *hsp-70p*::*mCherry* reporter. This resulted in strains PVH1 and PVH2 (see S3 Table).

For generation of PVH5 and PVH65, intestine-specific (*vha-6p*::*SID-1*::*unc-54 3′ UTR*) and muscle specific (*myo-3p*::*SID-1*::*unc-54 3′ UTR*) SID-1 constructs were microinjected into AM994 and integrated and backcrossed 5 times, before crossing into PVH2. The ability of tissue-specific SID-1 expression to allow tissue-specific knockdown by feeding RNAi bacteria was confirmed as described in [21,30].

To perform tissue-specific RNAi experiments in the *hsp-90*^int^ strain, which carries a *sid-1* (*pk3321*) mutation preventing transport of RNAi between tissues, *sid-1* was reintroduced under the control of a tissue-specific promoter to facilitate tissue-specific RNAi uptake. This was achieved by outcrossing the *hsp-90*^int^ (PVH2) strain into the PVH5 and PVH65 strains, which express integrated constructs of *sid-1* under the control of either the *myo-3* body wall muscle-specific promoter or the *vha-6* intestine-specific promoter respectively in a *sid-1 (pk3321)* background, resulting in strains PVH171 and PVH172 (see S3 Table).

### Confocal microscopy imaging

An inverted Zeiss LSM880 laser scanning confocal microscope was used to image *C*. *elegans*. Five worms were immobilized on 2% agarose pads using 5 mM levamisole and a coverslip. Images which were subsequently used to quantify fluorescence were taken using a 10× objective. Quantification of fluorescence was performed using ImageJ software as described in [30]. To image strain AM722 following heat shock, day 1 adults were then incubated at 35°C for 1 h followed by 3 h recovery at 20°C.

### RNA extraction, cDNA synthesis, and quantitative PCR (qPCR)

Nematodes were collected from NGM-Agar plates using chilled RNase-free water and worms were washed 3 times with RNase-free water to remove any residual bacteria (3 min, 500 × g). Excess water was removed and the pellet frozen at −80°C. TRIzol was added to samples before homogenization using a pellet grinder, following which RNA was extracted using a Direct-Zol RNA MiniPrep kit (Zymo Research, Cambridge Biosciences). RNA concentration was measured using a Thermo Scientific NanoDrop One, and 100 ng of RNA was reverse transcribed into cDNA using a Bio-Rad iScript cDNA synthesis kit. Quantitative PCR was performed using Bio-Rad Universal SYBR green Supermix in a Bio-Rad CFX Connect Real-Time System. Relative transcript expression for each gene was determined using the delta-delta C_t_ method as described previously [2,21].

Three biological replicates were performed per sample. Significance was determined using Student’s *t* test or one-way ANOVA with a cutoff of *p* < 0.05.

### Primers used for q RT PCR

*cdc-42 forw* 5′ TGTCGGTAAAACTTGTCTCCTG 3′

*cdc-42 rev* 5′ ATCCTAATGTGTATGGCTCGC 3′

*hsf-1 forw* 5′ GGACACAAATGGGCTCAATG 3′

*hsf-1 rev* 5′ CGCAAAAGTCTATTTCCAGCAC 3′

*hsp-70 forw* 5′ CGGTATTTATCAAAATGGAAAGGTT 3′

*hsp-70 rev* 5′ TACGAGCGGCTTGATCTTTT 3′

*hsp-90 forw* 5′ GACCAGAAACCCAGACGATATC 3′

*hsp-90 rev* 5′ GAAGAGCACGGAATTCAAGTTG 3′

### Lifespan assay

Animals were synchronized by egg-laying, and 100 L4-stage animals were selected per strain and transferred onto 5 NGM plates with 20 animals per plate. Every other day, each nematode was assessed for survival, or censorship and numbers were recorded. Animals were assessed as dead if they did not display movement when gently touched on the nose using a platinum wire and no pharyngeal pumping could be observed. Animals were censored if they crawled up the edge of the plate and became desiccated, burrowed into the agar, displayed the “bagging” phenotype where eggs hatched internally, displayed an exploded vulva phenotype, or if they could not be found. Any dead or censored animals were recorded and alive animals were transferred onto a new NGM plate. Data was analyzed using OASIS 2 online software [68] and GraphPad Prism. Assays were repeated twice (2 biological replicates) and changes in lifespan were considered statistically significant when *P < 0*.*05* after a Log-Rank test analysis.

### Thermotolerance assay

Strains were synchronized by egg-laying, with gravid adults allowed to lay eggs for 6 h and then removed from plates. Eggs were allowed to hatch and develop to L4 stage, at which point 3 replicate plates of 50 L4 animals were picked per condition per time point. The next day, plates containing day 1 adults were incubated at 35°C for either 6 h or at 37°C for 2 h; following which they were moved to 20°C. Animals were left to recover for 16 h at 20°C, then survival of animals was scored. Animals were scored as alive if movement or pharyngeal pumping was observed. The percentage of alive and dead animals were scored and mean survival rates were determined using Student’s *t* test. Three independent experiments were performed for each strain (*n* = 50 worms) and error bars indicate SEM.

### Transcriptomic profiling via RNA-seq

RNA was extracted from samples as described above. Agarose gel electrophoresis using a 1% gel was performed for a visual determination of sample quality, and RNA integrity number (RIN) was determined by the University of Leeds Next Generation Sequencing Facility using an Agilent 2200 TapeStation. RNA-seq was performed by Novogene (Hong Kong) on an Illumina Hi-Seq PE150 platform. Calculation of log_2_(fold change), *p* values and corrected *p* values were performed by Novogene. WBCel235 was used as the reference genome for annotation. GO term analysis was performed using the publicly available online tool DAVID Bioinformatics Resources 6.8.

### Forward genetic screen using EMS mutagenesis, whole-genome sequencing, and a Hawaiian *C*. *elegans* SNP mapping approach

*hsp-90*^int^ animals were synchronized by bleaching, allowed to develop to L4 stage, and incubated in 100 mM EMS solution for 4 h at 20°C. A total of 1,000 mutagenized adults (P_0_ generation) were allowed to lay eggs (F_1_ generation) on NGM plates overnight and removed the following day. F_1_ eggs were allowed to mature and also lay eggs (F_2_ generation), following which adult F_1_ animals were removed. F_2_ animals were grown to L4 stage and screened under a fluorescent microscope for the desired phenotype of visibly altered *hsp-70p*::*mCherry* reporter fluorescence. In total, approximately 20,000 F_2_ genomes were screened. Individuals identified by this method were isolated onto 35 mm plates and allowed to self-fertilize and progeny monitored to ensure homozygous phenotypes. This identified candidate mutant strains 1–4 showing reduced *hsp-70p*::*mCherry* reporter fluorescence.

To identify mutations in these candidate strains which were potentially causal for the phenotypes of visibly reduced *hsp-70p*::*mCherry* fluorescence (mutants 1–4), a *C*. *elegans* Hawaiian SNP mapping approach as described in Doitsidou and colleagues was used [34]. The candidate populations identified through phenotypic screening, as well as the control strains (N2, *hsp-90*^control^, and *hsp-90*^int^), were each outcrossed to the Hawaii/CB4856 alternative wild-type strain. At the F_2_ stage of each of these outcrosses, 50 F_2_ animals were isolated and allowed to self-fertilize, and the 50 heterozygous populations subsequently recombined into a single sample for each outcross. These samples were frozen as pellets, from which genomic DNA was subsequently extracted using a Gentra PureGene Tissue Kit. WGS of genomic DNA was performed by Novogene (Hong Kong) Company Limited on an Illumina Hi-Seq 2500 platform. WBCel235 was used as the reference genome for annotation.

Comparisons of data from each strain identified almost 15,000 SNPs as mutations. To determine which mutations were potentially causal for the phenotypes of interest, we used publicly available “CloudMap” workflows on the online data analysis platform Galaxy, which we followed according to the user guide available in the CloudMap data library. The “CloudMap Hawaiian Variant Mapping with WGS and Variant” workflow pipeline [69] was used to calculate the ratio of Bristol-derived to Hawaii-derived alleles at each SNP in each strain, and the “CloudMap Variant Discovery Mapping” workflow was used to remove SNPs that also occurred in control strains. Analysis was performed using the WormBase version WS266 that uses the WBcel235 reference genome. SNPs identified in this manner were taken forward as potentially causal if they had a Bristol-derived to Hawaii-derived allele ratio of less than 0.25 (S2 Table). The gene transcripts affected by these potential causal SNPs were identified using annotation performed by Novogene. Transcripts were excluded if they were classed as intergenic or pseudogenes.

### Developmental assay and egg laying rate

Nematodes were age-synchronized by transferring adult animals on to a new plate and worms were allowed to lay eggs for 2 h before removing. Embryos were grown at 20°C for 72 h (day 1 of adulthood) before assessment of their developmental stages. At least 3 independent experiments were performed (*n* > 100 worms each).

For the egg laying rate, 10 day 1 adult animals were allowed to lay eggs for 6 h and the average number of eggs laid per hour was calculated. At least 3 independent experiments were performed with *n* > 10 worms per replicate.

### Gene knockdown by RNAi

Populations were synchronized by egg-laying on HT115 *E*. *coli* transformed with appropriate RNAi vectors (J. Ahringer, University of Cambridge, Cambridge, United Kingdom) over 2 generations. Synchronized F_2_ generation eggs were allowed to develop and were used in experiments as day 1 adults.

## Supporting information

S1 Fig Development of *hsp-90*^*int*^ animals is unaffected. (**A**) Developmental stages at day 3 of life (72 h after hatching) in Wt (N2, Bristol), *hsp-90*^*control*^, *hsp-90*^*int*^, and *hsp-90*^*neuro*^ strains. (**B**) Average number of eggs laid per hour in Wt (N2) compared to *hsp-90*^*control*^, *hsp-90*^*int*^, and *hsp-90*^*neuro*^ strains. (**C**) Percentage of progeny that hatched as viable L1 larvae in Wt (N2) compared to *hsp-90*^*control*^, *hsp-90*^*int*^, and *hsp-90*^*neuro*^ strains. (A–C): Three replicates of 100 worms per strain. (**B**) Significance was determined using Student’s *t* test. (**A, C**) Significance was determined using one-way ANOVA. ***P < 0.001; ****P < 0.0001, **P < 0.01; n.s. = not significant. (**D**) Confocal images of body wall muscle cells. Age-synchronized day 1 adults expressing *myo-3p*::*GFP* (RW1596) and crossed into the genetic background of *hsp-90*^*control*^ *and hsp-90*^*int*^ were imaged. (**E**) Thermotolerance of control animals (AM722) and *hsp-90*^*int*^ allowing for muscle-specific RNAi (PVH171) and treated with *hsp-70* RNAi or empty vector (EV) RNAi, after a 2-h and 4-h HS at 37°C; *n* > 3 replicates of 50 animals per strain per time point. Significance was determined using two-way ANOVA. ****P < 0.0001; n.s. = not significant. Source data for S1A–S1C and S1E Fig is provided in S6 Data.
(PDF)

S2 Fig The TCS-induced transcriptional program is distinct from the HSF-1–mediated heat shock response. (**A**) Differential chaperone gene expression of *hsp-90*^*int*^ and *hsp-90*^*neuro*^ compared to *hsp-90*^*control*^ strain. Lists of differentially expressed chaperones in each strain were compared to known *C*. *elegans* chaperone genes [52]. log2 FC (fold-change) compared to the *hsp-90*^*control*^ strain. P_adj_ = Bonferroni corrected *P* value. (**B**) Venn diagram showing the overlap of 18 genes (1.7%) that are commonly up-regulated between a TCS-active strain (*hsp-90*^*int*^) at 20°C (this study) compared to N2 Bristol during HS [33]. *P* value overlap <0.04 was calculated using probability mass function of overlap size based on hypergeometric distribution.
(PDF)

S3 Fig *hsp-70p*::*mCherry* fluorescence intensity of *hsp-90*^*int*^ mutant strains isolated by EMS mutagenesis. (**A**) Confocal images of the 4 *hsp-90*^*int*^ mutant strains (mutant 1–4) showing reduced *hsp-70p*::*mCherry* expression in the body wall muscle compared to the parent strain *hsp-90*^*int*^. Scale bar = 100 μm. (**B**) Quantification of *hsp-70p*::*mCherry* fluorescence intensity in the EMS mutagenesis generated *hsp-90*^*int*^ mutant strains. *P < 0.05; **P < 0.01; ****P < 0.0001. Three biological replicates per image with 5 or more animals per replicate. Significance compared to mean fluorescence intensity in *hsp-90*^int^ was determined using Student’s *t* test. Source data is provided in S7 Data.
(PDF)

S4 Fig The *hsp-70* promoter contains a CEH-58 consensus sequence. (**A**) Motif scanning in the *hsp-70* promoter identifies a consensus motif for the homeobox transcription factor CEH-58 of NNTAATTRNN (CIS-BP database, [43]). The CEH-58 consensus motif is located 800 base pairs upstream of the first ATG (marked CEH-58, blue). Scanning also identified 2 canonical heat shock elements (HSEs, marked in red) of the form TTCNNGAA at 106 and 758 base pairs upstream of the ATG. (**B**) Venn diagram of differentially regulated genes in *hsp-90*^int^ containing CEH-58 and/or HSF-1 motifs in their promoter regions. (**C**) GO-term enrichment of 34 differentially regulated genes containing only CEH-58 motifs in their promoter. (**D**) GO-term enrichment of 12 differentially regulated genes containing both, HSF-1 and CEH-58 motifs. Expression of genes found in muscle cells are indicated in red. (**E**) GO-term enrichment of 125 differentially regulated genes containing only HSF-1 motifs in their promoter. Source data for S4C–S4E Fig is provided in S8 Data.
(PDF)

S5 Fig Extracellular peptides are involved in the regulation of the HSR and TCS. (**A**) Predicted extracellular peptides *txt-4* and *txt-8* are required in the muscle for TCS-mediated *hsp-70* induction during HS. Quantification of whole-animal *hsp-70* transcripts during muscle-specific RNAi (strain PVH171) against *txt-4*, *txt-5*, *txt-8*, *txt-12*, and *txt-14* compared to *EV* at 20°C and during HS (35°C). (**B**) Predicted extracellular peptides *txt-4*, *txt-5*, *txt-8*, *txt-12*, and *txt-14* are suppressors of TCS-mediated *hsp-70* induction in the intestine during HS. Quantification of whole-animal *hsp-70* transcripts during intestine-specific RNAi (strain PVH172) against *txt-4*, *txt-5*, *txt-8*, *txt-12*, and *txt-14* compared to *EV* at 20°C and during HS (35°C). (**C**) Systemic RNAi-mediated knockdown of *txt-4* and *txt-12* in a “TCS-inactive” control strain (AM722) reduces *hsp-70* expression at 35°C to approximately 25% compared to control RNAi (EV), whereas *txt-5* and *txt-8* RNAi reduce *hsp-70* levels to 75% compared to EV RNAi. (**D**) Quantification of whole-animal *hsp-70* transcripts during muscle-specific (PVH171), intestine-specific (PVH172), and systemic (AM722) *txt-4 or txt-8* RNAi and simultaneous *txt-4/txt-8* RNAi at 20°C. Error bars represent SEM of the 3 biological replicates. Significance compared to control RNAi (EV) was determined using two-way ANOVA. *P < 0.05; **P < 0.01; ***P < 0.001; ****P < 0.0001; n.s. = not significant. Quantification of *hsp-70p*::*mCherry* fluorescence during (**E**) systemic *hsf-1*, *txt-1*, and *ceh-58* RNAi (strain AM722); (**F**) muscle-specific *hsf-1*, *txt-1*, and *ceh-58* RNAi (strain PVH171); and (**G**) intestine-specific *hsf-1*, *txt-1*, and *ceh-58* RNAi (strain PVH172) compared to control RNAi (EV) at 20°C and after a 1-h HS at 35°C. (**E–G**) At least 5 animals per image; 1 biological replicate. (**H**) Quantification of *hsf-1* transcripts during *hsf-1*, *EV/hsf-1*, *hsf-1/txt-1*, and *hsf-1/ceh-58* RNAi compared to control (EV) RNAi in strain AM722 at 20°C and after a 1-h HS at 35°C. (**I**) *txt-1* transcripts during *txt-1*, *EV/txt-1*, *hsf-1/txt-1*, and *txt-1/ceh-58* RNAi compared to control (EV) RNAi in strain AM722 at 20°C and after a 1-h HS at 35°C. (**J**) *ceh-58* transcripts during *ceh-58*, *EV/ceh-58*, *hsf-1/ceh-58*, and *txt-1/ceh-58* RNAi compared to control (EV) RNAi in strain AM722 at 20°C and after a 1-h HS at 35°C. (**H–J**) Error bars represent SEM of the 3 biological replicates. Significance compared to control RNAi (EV) was determined using two-way ANOVA. * **** *P* < 0.0001; n.s. = not significant. Source data for S5A–S5J Fig is provided in S9 Data.
(PDF)

S1 Table Genes up-regulated in both TCS-activated strains, *hsp-90*^*int*^ and *hsp-90*^*neuro*^, compared to wild type (N2) and *hsp-90*^*control*^. The subcellular localization was predicted from the amino acid sequence using the DeepLoc 1.0 webtool (https://services.healthtech.dtu.dk/service.php?DeepLoc-1.0).
(PDF)

S2 Table A total of 45 SNPs (corresponding to 40 genes) identified by the mutagenesis screen and whole-genome-sequencing analysis. All phenotype-specific SNPs occurring in mutants 1–4 were ranked according to their CB4856: N2 ratio, with the lowest ratio of 0.139 ranked first. A ratio of 0, indicating 1 specific causal SNP was not identified. Multiple SNPs were identified in genes *Y48G1C*.*5*, *csk-1*, *ssp-19*, *sop-3* (gray shaded). LG, Position: chromosome and position of SNP on the genome. Reference genome WS266 or a combination of WS266 and WS220 were used to identify SNPs.
(PDF)

S3 Table *C*. *elegans* strains used in this study. (PDF)

S4 Table Candidate genes used for the tissue-specific RNAi screen (see Fig 4). (PDF)

S5 Table Data sheet of the motif analysis of differentially expressed genes in *hsp-90*^*int*.^ (XLSX)

S6 Table Differentially expressed genes in *hsp-90*^*int*.^ (XLSX)

S7 Table Differentially expressed genes in *hsp-90*^*neuro*^. (XLSX)

S1 Data Source data underlying Fig 1B, 1C, 1D, 1E and 1F. (XLSX)

S2 Data Source data underlying Fig 2D and 2E. (XLSX)

S3 Data Source data underlying Fig 3A–3D. (XLSX)

S4 Data Source data underlying Fig 4B and 4C. (XLSX)

S5 Data Source data underlying Fig 5A–5G. (XLSX)

S6 Data Source data underlying S1A–S1C and S1E Fig. (XLSX)

S7 Data Source data underlying S3B Fig. (XLSX)

S8 Data Source data underlying S4C–S4E Fig. (XLSX)

S9 Data Source data underlying S5A–S5J Fig. (XLSX)

## Abbreviations

GO: Gene Ontology
HSF-1: heat shock factor 1
HSR: heat shock response
RIN: RNA integrity number
RNAi: RNA interference
TCS: transcellular chaperone signaling
WGS: whole-genome sequencing
