# Supplementary figures and images for: Transcellular chaperone signaling is an intercellular stress-response distinct from the HSF-1–mediated heat shock response
Source: PLoS Biol. 2023 Feb 13;21(2):e3001605. doi: 10.1371/journal.pbio.3001605 (PMC9956597; doi:10.1371/journal.pbio.3001605)

Supplementary Figure 1

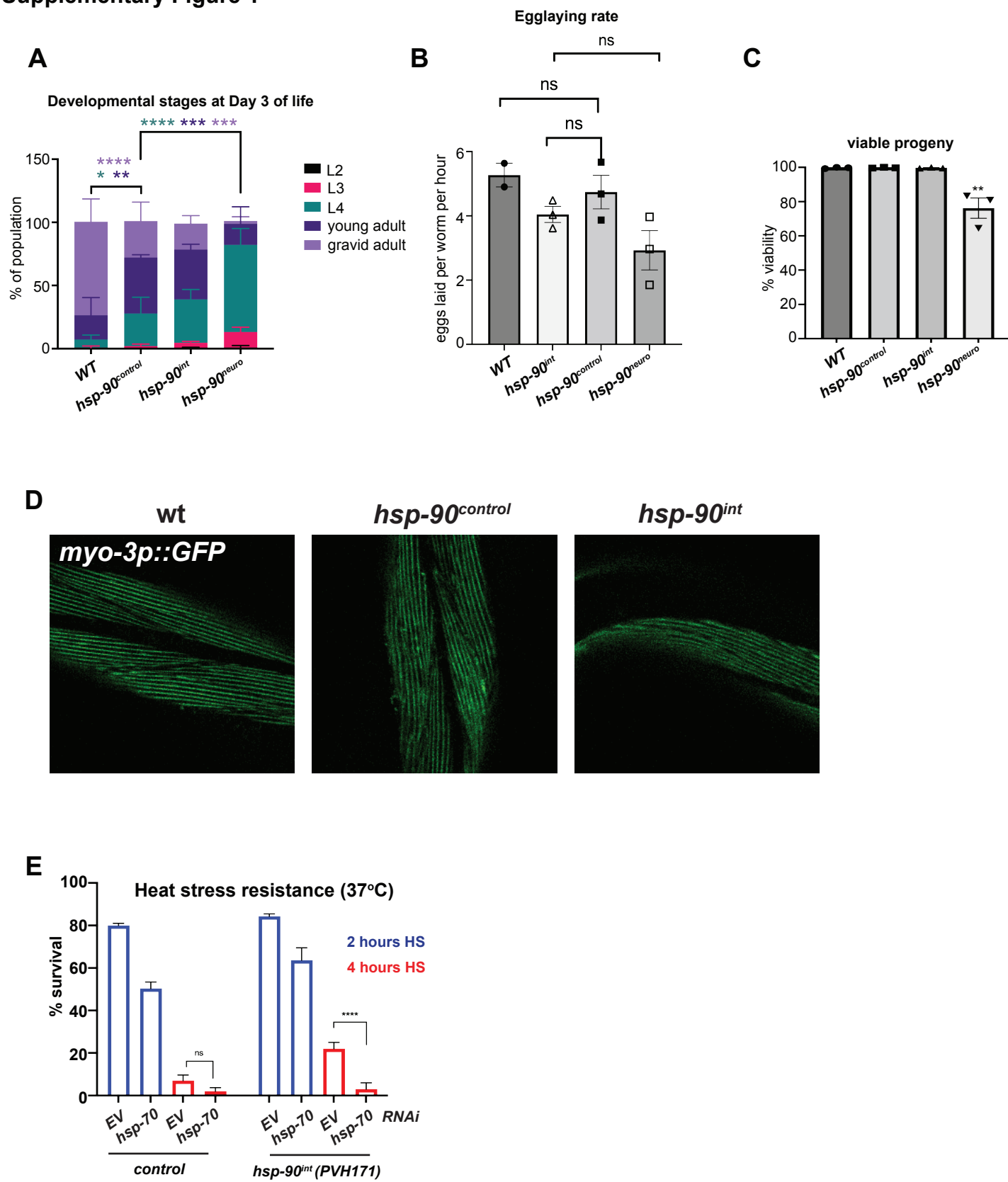

Supplement: S1 Fig — (A) Developmental stages at day 3 of life (72 h after hatching) in Wt (N2, Bristol), hsp-90control, hsp-90int, and hsp-90neuro strains. (B) Average number of eggs laid per hour in Wt (N2) compared to hsp-90control, hsp-90int, and hsp-90neuro strains. (C) Percentage of progeny that hatched as viable L1 larvae in Wt (N2) compared to hsp-90control, hsp-90int, and hsp-90neuro strains. (A–C): Three replicates of 100 worms per strain. (B) Significance was determined using Student’s t test. (A, C) Significance was determined using one-way ANOVA. ***P < 0.001; ****P < 0.0001, **P < 0.01; n.s. = not significant. (D) Confocal images of body wall muscle cells. Age-synchronized day 1 adults expressing myo-3p::GFP (RW1596) and crossed into the genetic background of hsp-90control and hsp-90int were imaged. (E) Thermotolerance of control animals (AM722) and hsp-90int allowing for muscle-specific RNAi (PVH171) and treated with hsp-70 RNAi or empty vector (EV) RNAi, after a 2-h and 4-h HS at 37°C; n > 3 replicates of 50 animals per strain per time point. Significance was determined using two-way ANOVA. ****P < 0.0001; n.s. = not significant. Source data for S1A–S1C and S1E Fig is provided in S6 Data. (PDF) [file pbio.3001605.s001.pdf]

Supplementary Figure 2

A

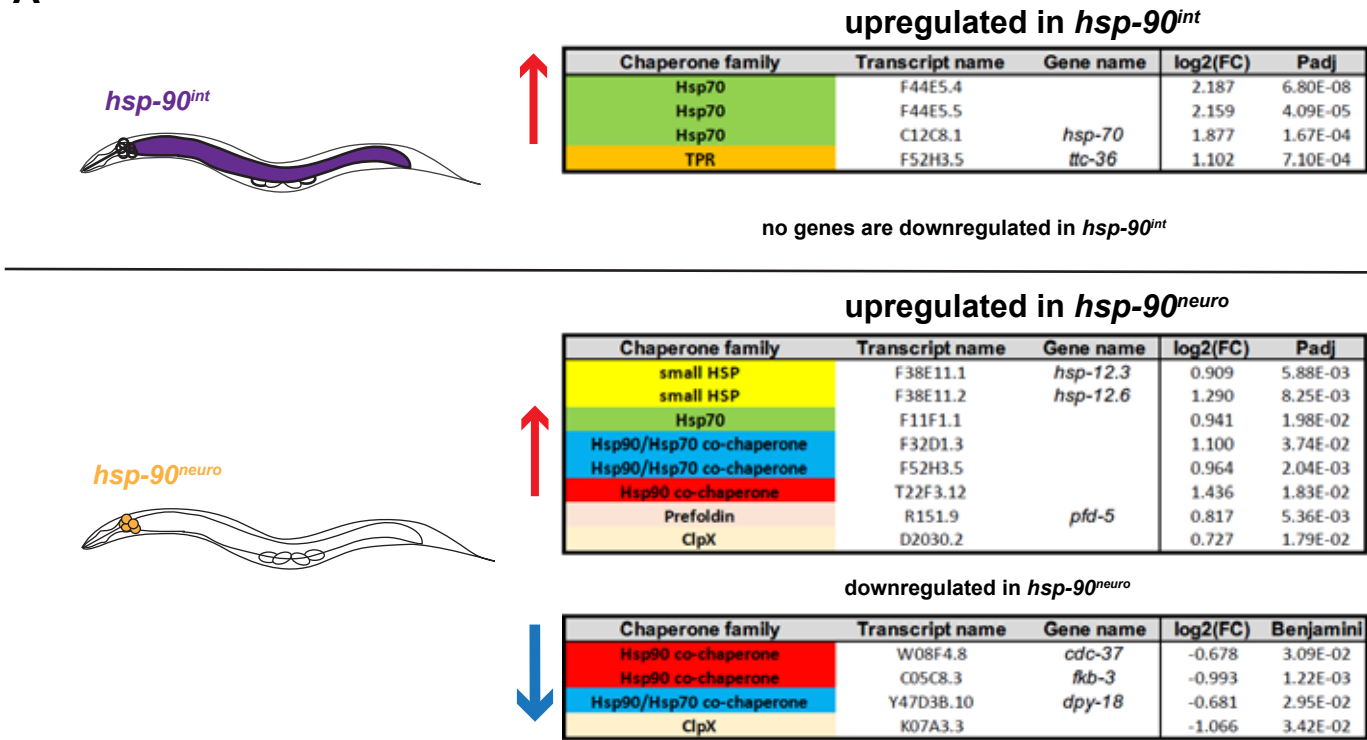

B

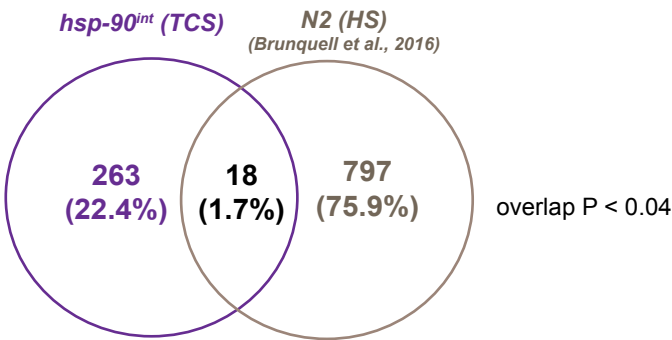

Supplement: S2 Fig — (A) Differential chaperone gene expression of hsp-90int and hsp-90neuro compared to hsp-90control strain. Lists of differentially expressed chaperones in each strain were compared to known C. elegans chaperone genes [52]. log2 FC (fold-change) compared to the hsp-90control strain. Padj = Bonferroni corrected P value. (B) Venn diagram showing the overlap of 18 genes (1.7%) that are commonly up-regulated between a TCS-active strain (hsp-90int) at 20°C (this study) compared to N2 Bristol during HS [33]. P value overlap <0.04 was calculated using probability mass function of overlap size based on hypergeometric distribution. (PDF) [file pbio.3001605.s002.pdf]

Supplementary Figure 3

A

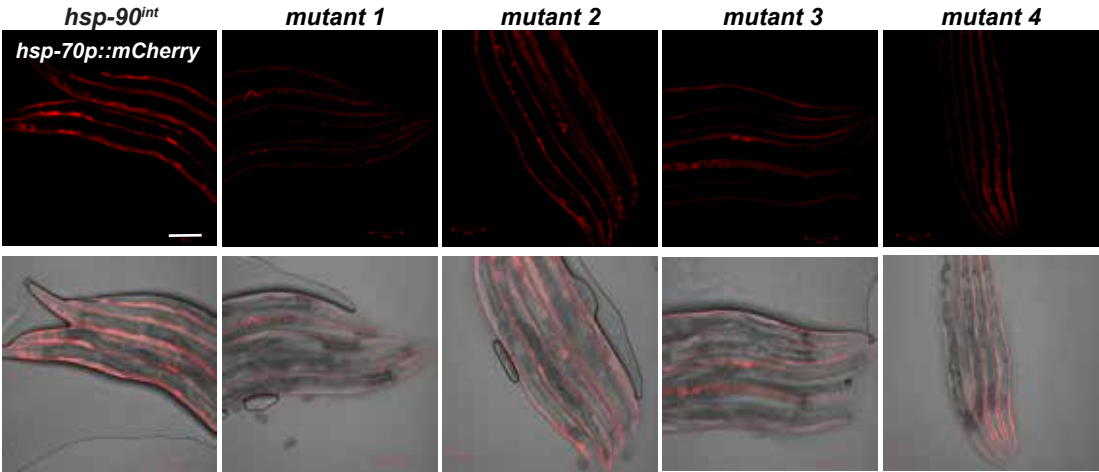

B

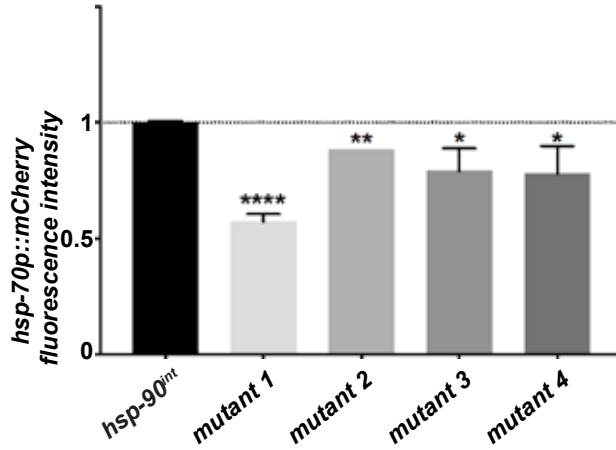

Supplement: S3 Fig — (A) Confocal images of the 4 hsp-90int mutant strains (mutant 1–4) showing reduced hsp-70p::mCherry expression in the body wall muscle compared to the parent strain hsp-90int. Scale bar = 100 μm. (B) Quantification of hsp-70p::mCherry fluorescence intensity in the EMS mutagenesis generated hsp-90int mutant strains. *P < 0.05; **P < 0.01; ****P < 0.0001. Three biological replicates per image with 5 or more animals per replicate. Significance compared to mean fluorescence intensity in hsp-90int was determined using Student’s t test. Source data is provided in S7 Data. (PDF) [file pbio.3001605.s003.pdf]

Supplemental Figure 4

A

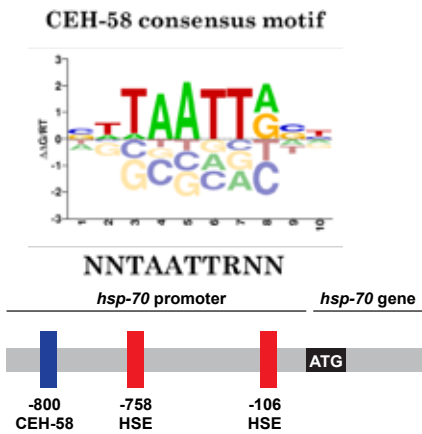

B

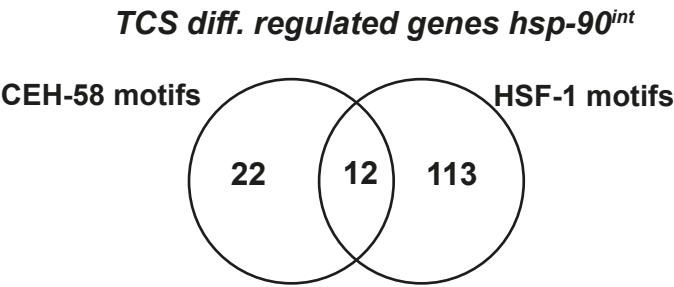

C

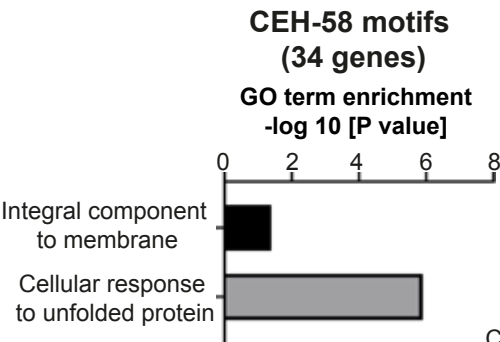

D

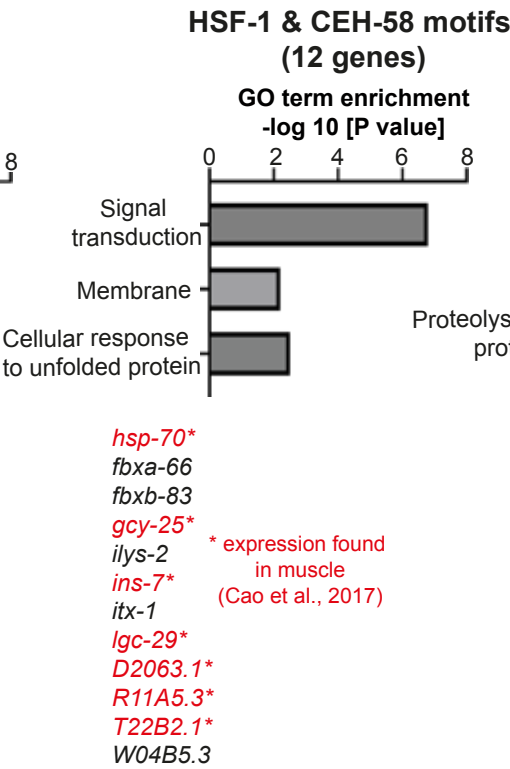

E

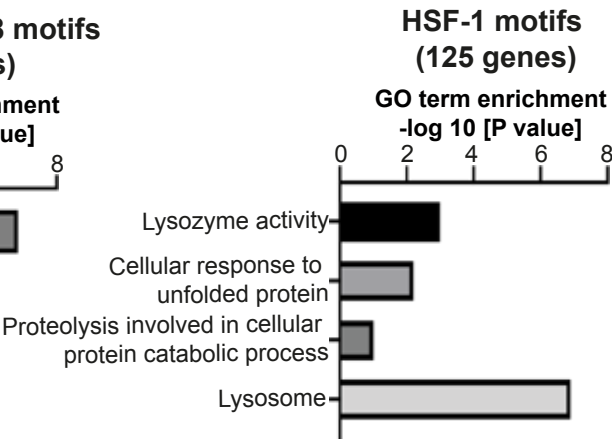

Supplement: S4 Fig — (A) Motif scanning in the hsp-70 promoter identifies a consensus motif for the homeobox transcription factor CEH-58 of NNTAATTRNN (CIS-BP database, [43]). The CEH-58 consensus motif is located 800 base pairs upstream of the first ATG (marked CEH-58, blue). Scanning also identified 2 canonical heat shock elements (HSEs, marked in red) of the form TTCNNGAA at 106 and 758 base pairs upstream of the ATG. (B) Venn diagram of differentially regulated genes in hsp-90int containing CEH-58 and/or HSF-1 motifs in their promoter regions. (C) GO-term enrichment of 34 differentially regulated genes containing only CEH-58 motifs in their promoter. (D) GO-term enrichment of 12 differentially regulated genes containing both, HSF-1 and CEH-58 motifs. Expression of genes found in muscle cells are indicated in red. (E) GO-term enrichment of 125 differentially regulated genes containing only HSF-1 motifs in their promoter. Source data for S4C–S4E Fig is provided in S8 Data. (PDF) [file pbio.3001605.s004.pdf]

Supplemental Figure 5

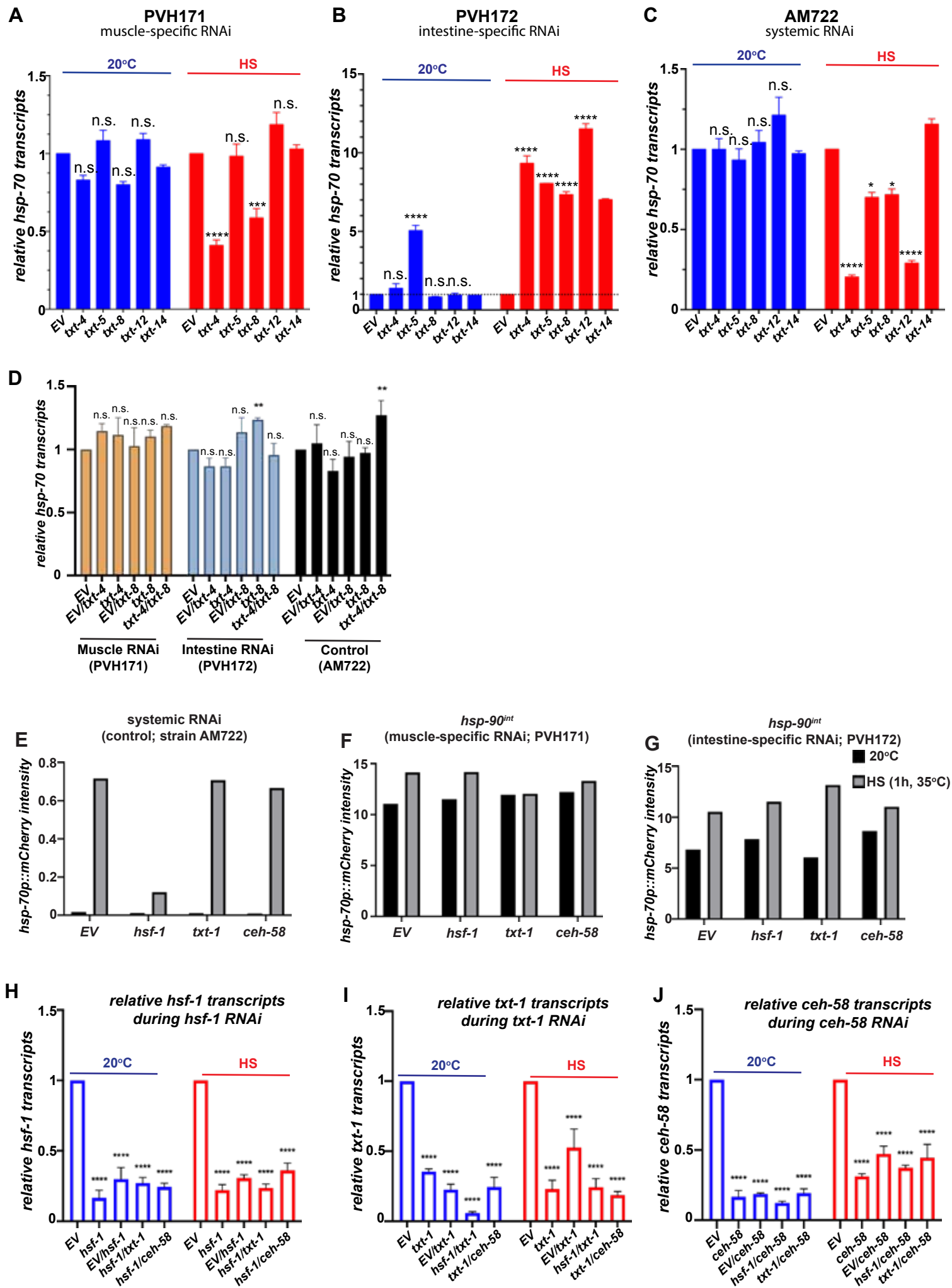

Supplement: S5 Fig — (A) Predicted extracellular peptides txt-4 and txt-8 are required in the muscle for TCS-mediated hsp-70 induction during HS. Quantification of whole-animal hsp-70 transcripts during muscle-specific RNAi (strain PVH171) against txt-4, txt-5, txt-8, txt-12, and txt-14 compared to EV at 20°C and during HS (35°C). (B) Predicted extracellular peptides txt-4, txt-5, txt-8, txt-12, and txt-14 are suppressors of TCS-mediated hsp-70 induction in the intestine during HS. Quantification of whole-animal hsp-70 transcripts during intestine-specific RNAi (strain PVH172) against txt-4, txt-5, txt-8, txt-12, and txt-14 compared to EV at 20°C and during HS (35°C). (C) Systemic RNAi-mediated knockdown of txt-4 and txt-12 in a “TCS-inactive” control strain (AM722) reduces hsp-70 expression at 35°C to approximately 25% compared to control RNAi (EV), whereas txt-5 and txt-8 RNAi reduce hsp-70 levels to 75% compared to EV RNAi. (D) Quantification of whole-animal hsp-70 transcripts during muscle-specific (PVH171), intestine-specific (PVH172), and systemic (AM722) txt-4 or txt-8 RNAi and simultaneous txt-4/txt-8 RNAi at 20°C. Error bars represent SEM of the 3 biological replicates. Significance compared to control RNAi (EV) was determined using two-way ANOVA. *P < 0.05; **P < 0.01; ***P < 0.001; ****P < 0.0001; n.s. = not significant. Quantification of hsp-70p::mCherry fluorescence during (E) systemic hsf-1, txt-1, and ceh-58 RNAi (strain AM722); (F) muscle-specific hsf-1, txt-1, and ceh-58 RNAi (strain PVH171); and (G) intestine-specific hsf-1, txt-1, and ceh-58 RNAi (strain PVH172) compared to control RNAi (EV) at 20°C and after a 1-h HS at 35°C. (E–G) At least 5 animals per image; 1 biological replicate. (H) Quantification of hsf-1 transcripts during hsf-1, EV/hsf-1, hsf-1/txt-1, and hsf-1/ceh-58 RNAi compared to control (EV) RNAi in strain AM722 at 20°C and after a 1-h HS at 35°C. (I) txt-1 transcripts during txt-1, EV/txt-1, hsf-1/txt-1, and txt-1/ceh-58 RNAi compared to control (EV) [file pbio.3001605.s005.pdf]
