# Supplementary material for: Transcellular chaperone signaling is an intercellular stress-response distinct from the HSF-1–mediated heat shock response
Source: PLoS Biol. 2023 Feb 13;21(2):e3001605. doi: 10.1371/journal.pbio.3001605 (PMC9956597; doi:10.1371/journal.pbio.3001605)
Supplement: S1 Table — The subcellular localization was predicted from the amino acid sequence using the DeepLoc 1.0 webtool (https://services.healthtech.dtu.dk/service.php?DeepLoc-1.0). (PDF) [file pbio.3001605.s006.pdf]

Transcript	Gene	txt gene	log FC *hsp-90* int	log FC *hsp-90neu*	Localisation	Predicted Human Orthologue	Function
F28H7.9	***sre-6***		2.617	2.485	Cell membrane		Class E serpentine receptor
F10D2.4	***str-112***		3.542	5.356	Cell membrane		Class R serpentine receptor
C05E4.14	***srh-2***		4.718	1.891	Cell membrane		Class H serpentine receptor
C07G3.2	***irg-1***		2.051	1.617	Cytoplasmic soluble		YbiA-like, NADAR, infection response
C06A1.2			1.017	0.962	ER membrane		Transmembrane
W05H12.1			3.300	1.508	ER membrane		NT signal peptide + non-cytoplasmic domain
Y19D10B.6			1.188	0.844	ER soluble		NT signal peptide + non-cytoplasmic domain
C01G10.16			2.769	1.939	Extracellular soluble		NT signal peptide
C02B8.12			2.158	1.635	Extracellular soluble		NT signal peptide
T21C12.3	***nlp-68***		3.436	1.031	Extracellular soluble	VMO1	Vitelline membrane outer layer protein I, N-termina signal peptide, carbohydrate binding sites
Y51A2D.14			1.663	0.928	Extracellular soluble		N-terminal signal peptide, alpha/beta hydrolase fold, fungal lipase-like domain
K10D11.1	***dod-17***		1.052	1.088	Extracellular soluble		Signal peptide, EGF-like, laminin EGF
C37C3.7			1.126	1.105	Extracellular soluble		CUB-like
ZK262.3			1.240	2.038	Extracellular soluble		NT signal peptide + non-cytoplasmic domain
Y9C9A.1			1.588	2.297	Extracellular soluble		NT signal peptide; cysteine-knot cytokine, prion-like domain-bearing protein
T20D4.5			2.839	1.739	Lysosome membrane		Galactose binding-like domain; peptide N glycanase, PAW domain; glycoprotein catabolism
Y38E10A.14			1.042	0.664	Nuclear soluble		

Supplementary Table 1. Gene hits shared between *hsp-90neuro hp-RNAi* and *hsp-90int hp-RNAi* strains, identified by RNA-Seq.
