# Supplementary material for: Transcellular chaperone signaling is an intercellular stress-response distinct from the HSF-1–mediated heat shock response
Source: PLoS Biol. 2023 Feb 13;21(2):e3001605. doi: 10.1371/journal.pbio.3001605 (PMC9956597; doi:10.1371/journal.pbio.3001605)
Supplement: S2 Table — All phenotype-specific SNPs occurring in mutants 1–4 were ranked according to their CB4856: N2 ratio, with the lowest ratio of 0.139 ranked first. A ratio of 0, indicating 1 specific causal SNP was not identified. Multiple SNPs were identified in genes Y48G1C.5, csk-1, ssp-19, sop-3 (gray shaded). LG, Position: chromosome and position of SNP on the genome. Reference genome WS266 or a combination of WS266 and WS220 were used to identify SNPs. (PDF) [file pbio.3001605.s007.pdf]

	LG	Position	CB4856:N2	Transcript	Mutant Strain
1	V	265899	0.139	*nhr-204*	3
2	II	5638240	0.148	*F59E12.3*	1
3	I	3115766	0.162	*C45E1.4*	3
4	II	14986715	0.162	*Y53F4B.6*	3
5	I	2773051	0.162	*sop-3*	3
6	I	2773053	0.162	*sop-3*	3
7	I	2886766	0.163	*arx-1*	1
8	V	4604780	0.171	*Y61A9LA.11*	2
9	III	10514651	0.175	*sly-1*	3
10	V	20362837	0.177	*K02E2.9*	1
11	V	17197315	0.177	*Y68A4A.5*	3
12	I	13867553	0.182	*taf-1*	3
13	IV	8981120	0.186	*C53B4.4*	3
14	I	1630320	0.187	*Y73E7A.1*	1, 3
15	II	416484	0.188	*C24H12.4*	1
16	IV	1997829	0.189	*xpc-1*	3
17	I	892612	0.190	*him-19*	2, 3
18	II	15240241	0.191	*Y46E12BL.2*	3
19	II	5580224	0.192	*T25E4.2*	1, 4
20	I	2563059	0.192	*ssp-19*	3
21	II	6879237	0.200	*C44B7.7*	4
22	I	2562717	0.200	*ssp-19*	1
23	V	6040440	0.200	*F29G9.1*	3
24	IV	14428193	0.200	*F55B11.5*	3
25	V	18960946	0.200	*ztf-20*	1
26	IV	15195137	0.200	*Y40H7A.15*	1
27	II	13372252	0.200	*Y48C3A.20*	1
28	IV	1180500	0.208	*Y104H12D.2*	3, 4
29	II	510660	0.208	*mab-9*	1
30	II	510660	0.208	*srh-105*	1
31	II	416454	0.210	*C24H12.4*	1, 3
32	I	67537	0.214	*csk-1*	3
33	I	67546	0.214	*csk-1*	3
34	I	67537	0.214	*Y48G1C.5*	3
35	I	67546	0.214	*Y48G1C.5*	3
36	X	197312	0.217	*T08D2.8*	3
37	X	21131	0.219	*Y35H6.3*	2
38	IV	2231205	0.227	*T04C4.1*	1
39	II	107742	0.231	*C50D2.3*	3
40	IV	2815036	0.231	*Y54G2A.13*	1
41	I	254848	0.238	*Y48G1BR.1*	1
42	I	62253	0.238	*Y48G1C.5*	4
43	I	36775	0.247	*Y74C9A.1*	1, 3
44	I	36775	0.247	*sesn-1*	1, 3
45	I	159472	0.250	*ptr-11*	1

Supplemental Table 2. 45 SNPs (corresponding to 40 genes) identified by the mutagenesis screen and whole-genome-sequencing analysis.
