# Supplementary material for: Transcellular chaperone signaling is an intercellular stress-response distinct from the HSF-1–mediated heat shock response
Source: PLoS Biol. 2023 Feb 13;21(2):e3001605. doi: 10.1371/journal.pbio.3001605 (PMC9956597; doi:10.1371/journal.pbio.3001605)
Supplement: S3 Table — (PDF) [file pbio.3001605.s008.pdf]

Strain	Referred to as	Genotype	Source
**N2**	Bristol		CGC
**CB4856**	Hawaii		CGC
**NL3321**		*sid-1* (pk3321)	CGC
**PS3551**		*hsf-1* (sy441) I	CGC
**AM722**		rmIs288 (*myo-2p::CFP*; *C12C8.1p::mCherry*)	Morimoto lab
**AM994**	*hsp-90* control	*sid-1* (pk3321); rmIs288 (*myo-2p::CFP*; *C12C8.1p::mCherry*)	Morimoto lab
**PVH2**	*hsp-90* int	*sid-1* (pk3321); rmIs288 (*myo-2p::CFP*; *C12C8.1p::mCherry*); pccIs002 (*vha-6p::daf-21 RNAi::unc-54 3'-UTR*)	this study
**PVH1**	*hsp-90* neu	*sid-1* (pk3321); rmIs288 (*myo-2p::CFP*; *C12C8.1p::mCherry*); pccIs001 (*F25B3.3p::daf-21 RNAi::unc-54 3'-UTR*)	this study
**PVH113**	mutant 1	*sid-1* (pk3321); rmIs288 (*myo-2p::CFP*; *C12C8.1p::mCherry*); pccIs002 (*vha-6p::daf-21 RNAi::unc-54 3'-UTR*); unknown EMS-induced mutations	this study
**PVH114**	mutant 2	*sid-1* (pk3321); rmIs288 (*myo-2p::CFP*; *C12C8.1p::mCherry*); pccIs002 (*vha-6p::daf-21 RNAi::unc-54 3'-UTR*); unknown EMS-induced mutations	this study
**PVH115**	mutant 3	*sid-1* (pk3321); rmIs288 (*myo-2p::CFP*; *C12C8.1p::mCherry*); pccIs002 (*vha-6p::daf-21 RNAi::unc-54 3'-UTR*); EMS-induced mutations	this study
**PVH116**	mutant 4	*sid-1* (pk3321); rmIs288 (*myo-2p::CFP*; *C12C8.1p::mCherry*); pccIs002 (*vha-6p::daf-21 RNAi::unc-54 3'-UTR*); EMS-induced mutations	this study
**PVH171**	*hsp-90int* ; muscle-specific RNAi	*sid-1* (pk3321); rmIs288; pccIs002; pccIs005 (*myo-3p::SID-1::unc-54 3'UTR*; *myo-2p::RFP*)	this study
**PVH172**	*hsp-90int*; Intestine-specific RNAi	*sid-1* (pk3321); rmIs288; pccIs002; pccIs004 (*vha-6p::SID-1::unc-54 3'UTR*; *myo-2p::RFP*)	this study
**PVH5**	muscle-specific RNAi	*sid-1* (pk3321); rmIs288; pccIs005 (*myo-3p::SID-1::unc-54 3'UTR*; *myo-2p::RFP*)	this study
**PVH73**	CB4856; N2	*CB4856;N2*	this study
**PVH67**	*hsp-90control;* CB4856	CB4856; sid-1 (pk3321); rmIs288 (myo-2p::CFP; C12C8.1p::mCherry)	this study
**PVH112**	*hsp-90int*; CB4856	CB4856*; sid-1* (pk3321); rmIs288 (*myo-2p::CFP*; *C12C8.1p::mCherry*); pccIs002 (*vha-6p::daf-21 RNAi::unc-54 3'-UTR*)	this study
**PVH119**	mutant 1 (EMS)	mutant 1; CB4856*; sid-1* (pk3321); rmIs288 (*myo-2p::CFP*; *C12C8.1p::mCherry*); pccIs002 (*vha-6p::daf-21 RNAi::unc-54 3'-UTR*)	this study
**PVH120**	mutant 2 (EMS)	mutant 2; CB4856*; sid-1* (pk3321); rmIs288 (*myo-2p::CFP*; *C12C8.1p::mCherry*); pccIs002 (*vha-6p::daf-21 RNAi::unc-54 3'-UTR*)	this study
**PVH121**	mutant 3 (EMS)	mutant 3; CB4856*; sid-1* (pk3321); rmIs288 (*myo-2p::CFP*; *C12C8.1p::mCherry*); pccIs002 (*vha-6p::daf-21 RNAi::unc-54 3'-UTR*)	this study
**PVH122**	mutant 4 (EMS)	mutant 4; CB4856*; sid-1* (pk3321); rmIs288 (*myo-2p::CFP*; *C12C8.1p::mCherry*); pccIs002 (*vha-6p::daf-21 RNAi::unc-54 3'-UTR*)	this study
**PVH65**	Intestine-specific RNAi	*sid-1* (pk3321); rmIs288; pccIs004 (*vha-6p::SID-1::unc-54 3'UTR*; *myo-2p::RFP*)	this study

Supplemental Table 3. *C. elegans* strains used in this study.
