# Supplementary material for: Transcellular chaperone signaling is an intercellular stress-response distinct from the HSF-1–mediated heat shock response
Source: PLoS Biol. 2023 Feb 13;21(2):e3001605. doi: 10.1371/journal.pbio.3001605 (PMC9956597; doi:10.1371/journal.pbio.3001605)
Supplement: S4 Table — (PDF) [file pbio.3001605.s009.pdf]

Gene	dataset
dod-17	RNA-Seq
mct-6	C50D2.3 partner
nhr-204	WGS
irg-1	RNA-seq
K03H6.2	WGS
C53B4.4	WGS
C44B7.7	WGS
T20D4.5	RNA-seq
ztf-20	WGS
ssp-19	WGS
C45E1.4	WGS
dlat-1	C50D2.3 partner
F29G9.1	WGS
Y48G1C.5	WGS
ceh-58	C50D2.3 partner
sly-1	WGS
arx-1	WGS
btbd-10	C50D2.3 partner
C06A1.2	RNA-seq
taf-1	WGS
nlp-68	RNA-seq
sesn-1	WGS
ptr-11	WGS
mab-9	WGS
sre-6	RNA-seq
C01G10.16	RNA-seq
F59E12.3	WGS
str-112	RNA-seq
Y54G2A.13	WGS
hsp-90	ctrl (proteostasis modulator)
C24H12.4	WGS
swsn-9	C50D2.3 partner
Y48C3A.20	WGS
Y46E12BL.2	WGS
Y51A2D.14	WGS
hsp-70	ctrl (proteostasis modulator)
T25E4.2	WGS
csk-1	WGS
Y61A9LA.11	WGS
Y9C9A.1	RNA-seq
C37C3.7	RNA-seq
srh-105	WGS
ZK262.3	RNA-seq
C32D5.1	C50D2.3 partner
Y68A4A.5	WGS
hsf-1	ctrl (proteostasis modulator)
T04C4.1	WGS
srh-2	RNA-seq
C50D2.3	WGS
tsct-1	C50D2.3 partner
Y74C9A.1	WGS
rack-1	C50D2.3 partner
skn-1	proteostasis modulator
pha-4	proteostasis modulator
pqm-1	proteostasis modulator
daf-16	proteostasis modulator

Supplemental Table 4. Candidate genes used for the tissue-specific RNAi screen (see Figure 4).
